# Structural Model for Recruitment of RIT1 to the LZTR1 E3 Ligase: Evidences from an Integrated Computational Approach

**DOI:** 10.1021/acs.jcim.1c00296

**Published:** 2021-04-01

**Authors:** Antonella Paladino, Fulvio D’Angelo, Teresa Maria Rosaria Noviello, Antonio Iavarone, Michele Ceccarelli

**Affiliations:** † BIOGEM Istituto di Ricerche Genetiche “G. Salvatore”, via Camporeale, Ariano Irpino 83031, Italy; ‡ Institute for Cancer Genetics, Columbia University, 1130 St Nicholas Ave, New York, New York 10032, United States; § Department of Electrical Engineering and Information Technology (DIETI), University of Naples “Federico II”, Via Claudio 21, Naples 80128, Italy; ∥ Department of Pathology and Cell Biology, Columbia University Medical Center, 1130 St Nicholas Ave, New York , New York 10032 United States; ⊥ Department of Neurology, Columbia University Medical Center, 1130 St Nicholas Ave, New York, New York 10032, United States

## Abstract

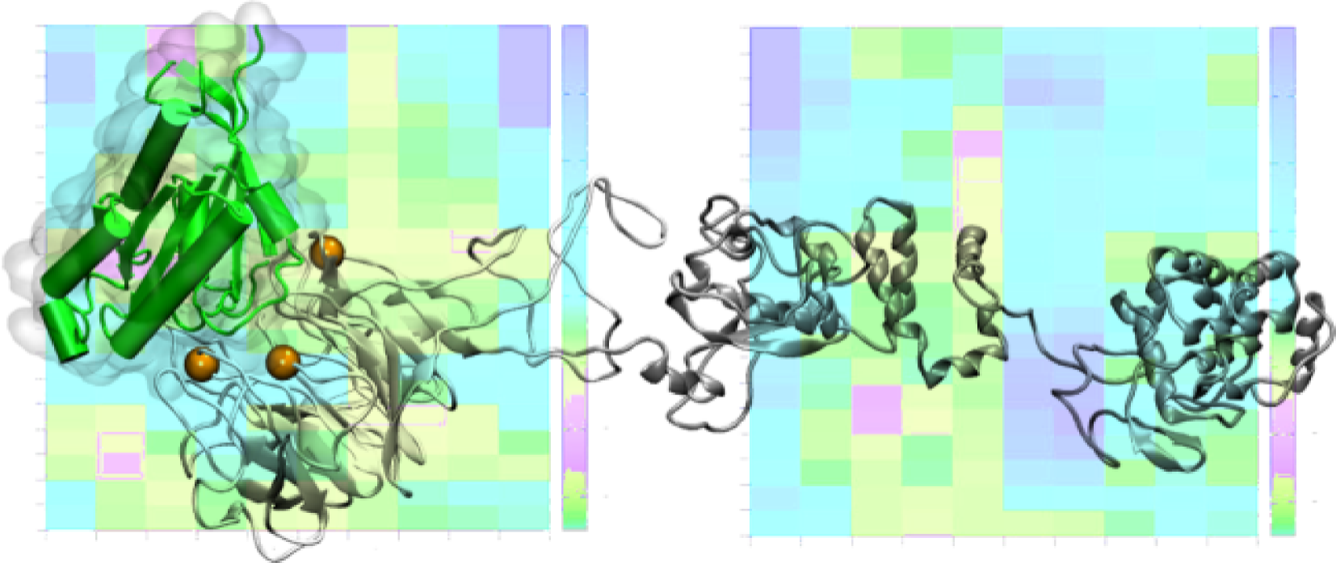

Leucine-zipper transcription regulator 1 (LZTR1) is a highly mutated tumor suppressor gene, involved in the pathogenesis of several cancer types and developmental disorders. In proteasomal degradation, it acts as an adaptor protein responsible for the recognition and recruitment of substrates to be ubiquitinated in Cullin3-RING ligase E3 (CRL3) machinery. LZTR1 belongs to the BTB-Kelch family, a multi-domain protein where the Kelch propeller plays as the substrate recognition region and for which no experimental structure has been solved. Recently, large effort mutational analyses pointed to the role of disease-associated LZTR1 mutations in the RAS/MAPK signaling pathway and RIT1, a small Ras-related GTPase protein, has been identified by mass spectroscopy to interact with LZTR1. Hence, a better understanding of native structure, molecular mechanism, and substrate specificity would help clarifying the role of LZTR1 in pathological diseases, thus promoting advancement in the development of novel therapeutic strategies. Here, we address the interaction model between adaptor LZTR1 and substrate RIT1 by applying an integrated computational approach, including molecular modeling and docking techniques. We observe that the interaction model LZTR1-RIT1 is stabilized by an electrostatic bond network established between the two protein surfaces, which is reminiscent of homologous ubiquitin ligases complexes. Then, running MD simulations, we characterize differential conformational dynamics of the multi-domain LZTR1, offering interesting implications on the mechanistic role of specific point mutations. We identify G248R and R283Q as damaging mutations involved in the recognition process of the substrate RIT1 and R412C as a possible allosteric mutation from the Kelch to the C-term BTB-domain. Our findings provide important structural insights on targeting CRL3s for drug discovery.

## Introduction

Glioblastoma (GBM) is the most common primary intrinsic malignant brain tumor; the identification of genetic alterations that drive the tumor initiation and progression, together with the functional consequences, is crucial to develop effective therapies.

LZTR1 (leucine-zipper transcription regulator 1) is detected as a mutational cancer driver gene in GBM.^1^ A recent comprehensive molecular characterization of ubiquitin pathway from 9125 tumor samples across 33 cancer types also found that LZTR1 is among the frequently mutated genes.^1,2^ Further, heterozygous germline loss-of-function LZTR1 variants have been linked to Schwannomatosis whereas rare variants of LZTR1 were identified in individuals with Noonan syndrome (NS), a development disorder, caused by mutations in components of the RAS/MAPK signaling pathway (i.e., RASopathies).^3−5^

Consistently, LZTR1 has been found by mass spectroscopy to interact with a Ras-related small GTPase, RIT1, an oncoprotein highly involved in Noonan syndrome and cancer. McCormick and collaborators show that, under physiological conditions, LZTR1 promotes RIT1 proteolysis through CUL3-mediated proteosomal degradation while pathogenic mutations affecting RIT1 or LZTR1 lead to RIT1 stabilization and contribute to hyperactivation of MAPK signaling.^6^

The central role of LZTR1 in the ubiquitination process and in the pathogenesis of several disorders renders this protein a hot therapeutic target. In light of direct structure–activity relationships, a deeper knowledge of its molecular shape and the definition of molecular parameters characteristic of the protein–protein interaction are essential points. All this information can serve to discriminate among different molecular states, to identify the “active” form of the tumor suppressor capable of substrate recruitment, and ultimately, to foresee other potential interactors.

The ubiquitin proteasome system is one of the major regulatory tools in cellular pathways: prior to proteasomal degradation, ubiquitination is mediated by a protein complex denoted as E3 ligase that simultaneously binds the protein substrate and the ubiquitin conjugating enzyme E2.^7^ The largest known class of ubiquitin complexes, Cullin-RING ligases (CRLs) are multi-subunit E3 ubiquitin ligases that recruit substrate-specific adaptors to catalyze protein ubiquitination. Cullin is a scaffold protein with the catalytic region at the C-terminus, able to interact with both E2 and ubiquitin, and an N-terminus that recruits a variety of receptor proteins and confers substrate specificity.^8^

In the present assembly, Cullin3 (Cul3)-based ubiquitin E3 ligase complexes catalyze the transfer of ubiquitin from an E2 enzyme to target substrate proteins: the C-terminal region of Cul3 binds Rbx1/E2-ubiquitin (a RING-domain protein that in turn recruits the E2 enzyme), while the N-terminal region interacts with various BTB (bric-à-brac, tramtrack, broad complex) domain proteins that serve as substrate adaptors.^9,10^

Then, the CUL3 adaptors recruit substrates via domains such as Kelch or MATH and they interact with Cullin via their BTB domain, a versatile protein–protein interaction domain also found in zinc-finger transcription factors. It has been shown that the SPOP BTB motif can mediate homodimerization and eventually heterodimerization; therefore, it is able to enroll two CUL3 subunits into the CRL3 complex.^11^ On the other side, the Kelch domain is the most widespread of the CRL3 substrate-recognition domains and recruits a diverse range of substrates.^11−16^ Despite different binding features and poor sequence conservation, a large three-dimensional similarity represents the key trait of E3-ligase substrate variety.

Lztr1 encodes a protein belonging to the BTB-Kelch superfamily, and it is involved in apoptosis and ubiquitination as a substrate adaptor in Cullin 3 (CUL3) ubiquitin ligase complexes. From a structural perspective, LZTR1 is an atypical member of the BTB-Kelch protein family, with an N-terminal β-propeller Kelch domain followed by two BTB-Back domains, contrarily to other members of the BTB-Kelch group, that present one BTB motif at the N-term and a Kelch domain at the C-term. Kelch proteins play a crucial role in organizing and adapting the multi-molecular complex between the enzyme Cullin3-dependent E3 ubiquitination ligase and the substrate that needs to be ubiquitinated and then degraded by the proteasome machinery.

In particular, the β-propeller serves as a substrate recognition domain, with each Kelch repeat domain binding a wide range of substrates. Although BTB-Kelch proteins form the largest subfamily of Cullin-RING E3 ligases (CRL3), their substrate complexes are mapped and structurally characterized only for KEAP1, KLHL3, and KLHL20.^14,17−20^

The lack of structural details on Kelch proteins and the number and diversity of potential physiological partners have limited the identification of molecular determinants either common to the whole superfamily or specific to each species.

Furthermore, a variety of experimental and theoretical evidences pointed to the role of conformational plasticity and structural adaptability displayed by the components of several CRL multi-complexes.^21−27^ Productive ubiquitination is a well-known dynamic process that tightly depends on the concerted coordination among all elements of the proteasomal machinery: the intrinsic flexibility of individual molecules, post-translational modifications, and the nature of protein–protein interactions contribute at various extent to optimal activities of CRLs.

Applying an integrated computational protocol, here we present the structural model of the binding between the substrate-binding protein LZTR1 and its substrate RIT1. Prior to the docking experiments, comparative modeling has been applied to produce the full-length three-dimensional structure of the multi-domain LZTR1. In addition, extensive all-atom molecular dynamics studies highlight the conformational variability of the substrate-binding protein in the complex LZTR1-RIT1: the investigation of the chemical–physical nature of this mode of interaction could favor an important step toward the rational discrimination among multiple substrate candidates, providing a mechanistic hypothesis on the role of pathogenic mutations in the elicitation of E3 ubiquitin activity and promoting the design of small molecule mimicry approaches to disrupt protein–protein interactions (PPI). Further, evaluating the modulation of the internal dynamics and flexibility of this multi-module complex could reveal coupled motions throughout the protein domains that facilitate the conformational change required for function.

## Results and Discussion

### LZTR1 3D Structure by Homology Modeling

No experimental three-dimensional structure is available, neither for the full-length multi-domain LZTR1 nor for its individual domains. Sequence alignments identified in the LZTR1 sequence a Kelch six-bladed β-propeller motif at the N-terminus and two BTB-Back domains at the C-terminus. Moreover, while several theoretical efforts have been made to model single domain LZTR1 and reproduce the structural interaction between BTB-Back and Cullin 3, this is the very first attempt to characterize the full-length model structure aimed at investigating the Kelch recognition mechanism and its mode of interaction with E3 ligase substrates.^1,3,6,28,29^

Sequence conservation analysis displays a helpful structural match to several substrate binding proteins containing the Kelch motif and BTB-Back domains variously associated to the E3 ligase machinery. Nevertheless, no full-length match is available for comparative modeling and a multi-template approach is used to build the LZTR1 homology model.

LZTR1 has a unique structural peculiarity compared to other BTB-Kelch family members: here an N-terminal Kelch domain is followed by two BTB domains, and both kinds of domains are known to mediate protein–protein interactions.

Homology models of the Kelch domain and the two BTB-Back domains of human LZTR1 are here generated using a comparative protein modeling approach as described in the Methods section (Figures S1 and S2). Comparative modeling produces a symmetric Kelch domain composed by six blades, circularly arranged around a central axis to make the β-propeller. Each blade is formed by four-stranded, twisted anti-parallel β-sheets (aka Kelch repeats), with the C-terminal βA strand closing the propeller by completing blade I. Disordered loop 325–385 connecting blades V and VI extends out of the Kelch domain providing a spatial link to the following BTB domain.

**Figure 1:**
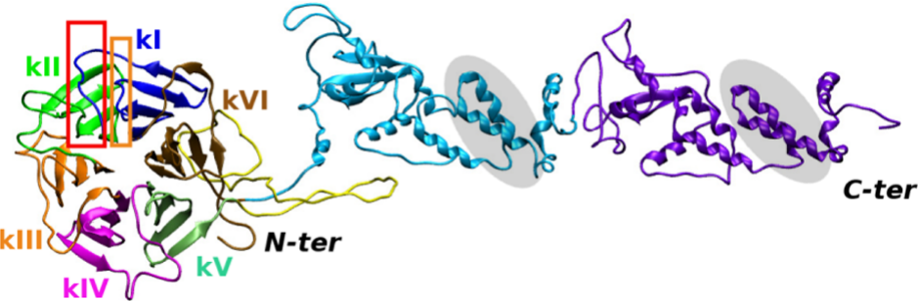
Homology modeling of the human LZTR1. A three-dimensional structure is shown in cartoon representation. The N-terminal Kelch repeats (kI-VI) are colored and labeled. Orange and red boxes indicate BC and DA loops, respectively. BTB1-back and BTB2-back are rendered in cyan and violet cartoons. The long disordered loop within the Kelch domain (∼325–385 ) is displayed in yellow. The 3-box within the back domain is indicated with transparent gray ellipses.

At one face of the propeller, loops joining β-strands are known to mediate the protein–protein interaction. In particular, DA (from adjacent blades) and BC loops at the outer face of the Kelch shape the binding area by exposing amino acids for the recognition and recruitment of substrates (Figure 1).

BTB-Back scaffold consists of a cluster of α-helices flanked by short β-sheet. In detail, the C-terminal domain comprises five α-helices, namely, α7-α11, with α11 residues contributing most of the interactions at the dimerization interface.^11^ In addition, it has been shown that BTB1-Back is required for dimerization and that Cul3 interactions could be mediated by both BTB-Back domains with 3-box (α7-α8) of the BTB-Back providing the surface binding for Cul3 (Figure 1 and Figure S1).^1,6^

In order to overcome steric clashes and optimize structural constraints, prior to protein–protein interaction studies, our LZTR1 model structure is refined by a full cycle of energy minimization and equilibration and subjected to MD simulation studies in an explicit solvent (see Methods).

From Figure 1, LZTR1 folds into an elongated structure made by three well-separated structural blocks. Apart from assessing the reliability of our LZTR1 3D model, MD studies highlight large conformational flexibility and a significant mutual rearrangement of the three domains, with no expense to intrinsic structural stability: the evolution of protein secondary structures and root mean square deviations (RMSD) along the simulation time confirm the structural stability of both LZTR1 secondary and tertiary structures (Figure S3).

### LZTR1-RIT1 Docking Model: Stability and Protein–Protein Interactions (PPI)

Next, we planned to investigate the recruitment mechanism used by LZTR1 to identify and bind RIT1. To this aim, we used a stepwise approach that preliminarily identified the putative interaction regions between the proteins based on the distribution of disease-related mutants. Indeed, recent research evidenced that RIT1 recognizes and binds LZTR1 at the Kelch domain, and pathogenic mutations, either in LZTR1 or RIT1, have proven capable to impair RIT1 degradation, confirming a functional interaction and redundancy.^6^ Then, also exploiting this information, we performed docking experiments.

On the other side, involved in the RAS-MAPK signaling pathway, RIT1 is a small GTP-binding protein (219 amino acids) with a characteristic βαβ fold (Rossmann fold) that exchanges between inactive (GDP) and active (GTP) forms to regulate cell survival. The majority of oncogenic mutations in RIT1 are localized near the switch-II domain (between β3 and α2), whose structural transitions are essential for its GTPase activity. In particular, immunoblots of HEK293T cells transfected with a panel of GST-tagged RIT1 mutants were produced to verify the interaction with endogenous LZTR1. Namely, none of the A57G, A77P, E81G, F82L, T83P, Y89H, and M90I mutants showed detectable binding to LZTR1. Mapping of these mutations on the protein surface indicates their preferential clustering on one face of the small Ras-domain (Figure S4). Conversely, most of the LZTR1 point mutations involved in cancer, NS, and Schwannomatosis are clustered at the Kelch repeats.^1,3,5,28^ More importantly, besides preventing RIT1 degradation, LZTR1 mutants, S247N and R284C, were shown to decrease interaction with RIT1, thus suggesting a physical participation of the kelch-IV repeat in the recognition mechanism.^6^

Several efforts have been dedicated to understand the effects of pathological mutations spread all over the LZTR1 structure. Previous works showed net clustering of point mutations on the Kelch domain with loss of function as the main mechanism. In particular, spatial localization of missense alterations points at a functional role in the RAS/MAPK signaling of LZTR1 via direct interaction with RAS proteins.^1,3,5,28,30,31^ Based on these evidences, we collected known missense LZTR1 somatic and germline mutations listed in public databases (Cosmic, TCGA, and ClinVar) and predicted their pathogenicity index (see Methods), thus discriminating between tolerated, mildly, and highly damaging (scored 1 to 4 with increasing pathogenicity). The full list of 252 non-synonymous missense variations covering the full-length LZTR1 sequence is given in the Supporting Information (Table S1).

**Figure 2:**
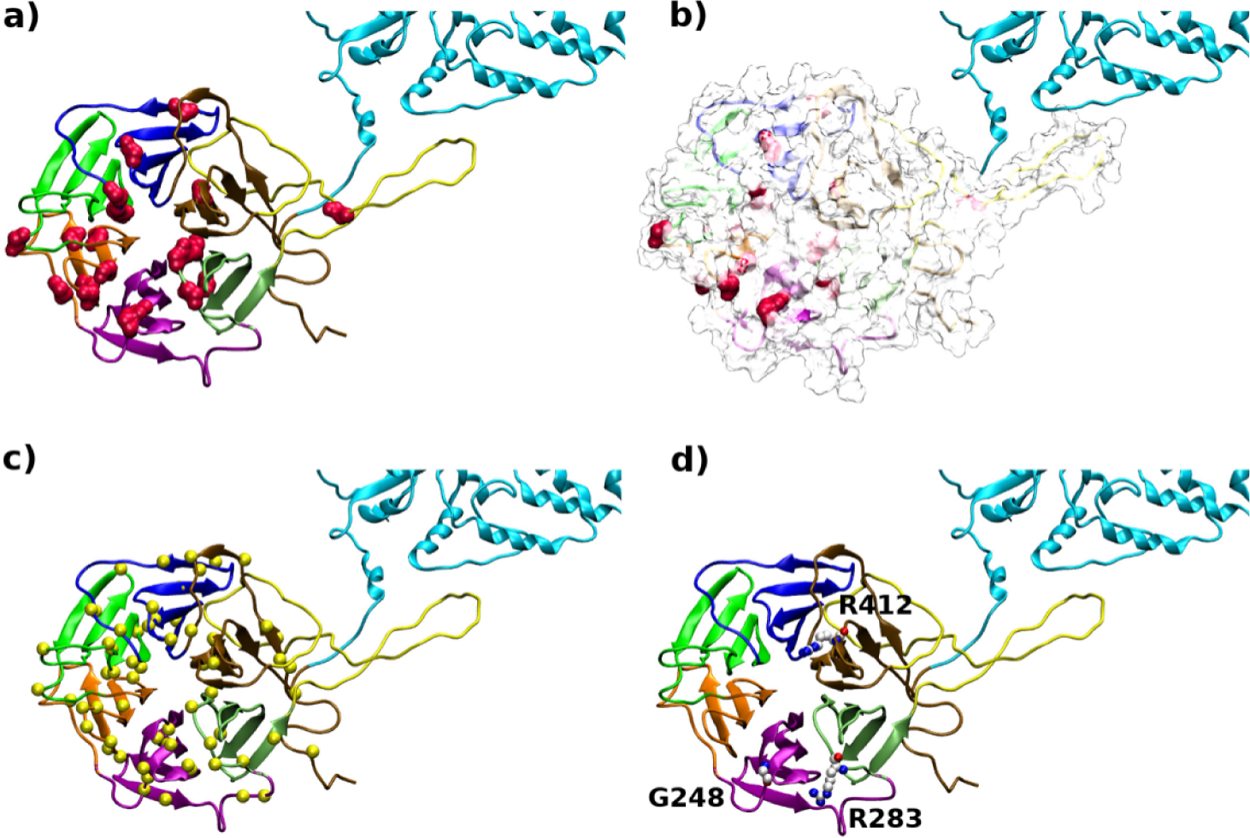
Spatial distribution of pathogenic mutations. Zoom-in the LZTR1 Kelch domain variants: (a, b) point mutations from refs (1)(3)(5)(29), and (32) are displayed as red backbone atoms; in (b) Kelch domain is shown in ghost surface; (c) pathogenic point mutations from deep mutational scanning analysis are rendered as yellow spheres (see Table S1); (d) mutated amino acids G248 and R283 (k-IV) and R412 (k-VI) are indicated as white CPK. Kelch repeats are colored as in Figure 1.

From a close examination of LZTR1 mutants, we observed that point mutations span the whole protein sequence, with a larger proportion at the N-terminal Kelch domain (141/252 = ∼56%), most of which are predicted to be highly damaging (73/131 = ∼56%). In the present study, we focused on missense germline variants, which have been related to inherited disorder with multiple schwannomas, NS, and GBM (Figure 2, Table 1, Figure S5, and Table S1) and might take a key part in substrate binding.

**Table 1:** Lztr1 Germline Mutations^a^

missense germline	condition
**R68S**	LZTR1-related
**H120Q**	NS
**H121D**	NS
**R170Q**^b^	*n.a*
**S247N**	NS
**G248R**	NS/Schw 2
**R283Q**	NS/conflicting
**R284C**	NS/Schw 2
**R412C**	NS/Schw 2
**A465V**^c^	*n.a.*
P520L	Schw 2
Y529H	NS
P635L	NS
R688C	Schw 2
Y749H	NS
R755Q	NS

a Germline missense mutations of the LZTR1 coding region associated to annotated (and verified) human phenotypes are retrieved from the ClinVar archive. Mutations within the Kelch domain are listed in bold.

b Mutation from ref (6).

c Mutation data from The Cancer Genome Atlas (TCGA). NS: Noonan syndrome; Schw 2: Schwannomatosis 2.

From Figure 2, missense mutations not only concern superficial amino acids and 73 highly predicted damaging variants (yellow spots in Figure 2c) are randomly distributed all over the repeats.

We therefore decided to investigate the role of specific mutations in the recognition mechanism triggered by LZTR1.

In detail, we used a combination of docking approaches to identify the best interaction model that fulfill all structural requirements and experimental information available (see Methods).

Distinct fragments of the six-bladed Kelch have been identified for substrate recognition and recruitment in other BTB-Kelch proteins, namely, BC and DA loops within the same or across adjacent repeats on the top face of the propeller (Figure 1).^14,16,20,33^ Moreover, RIT1 mutational data represent a valid tool to guide (when allowed in the docking method) and select the best protein–protein complex.

**Figure 3:**
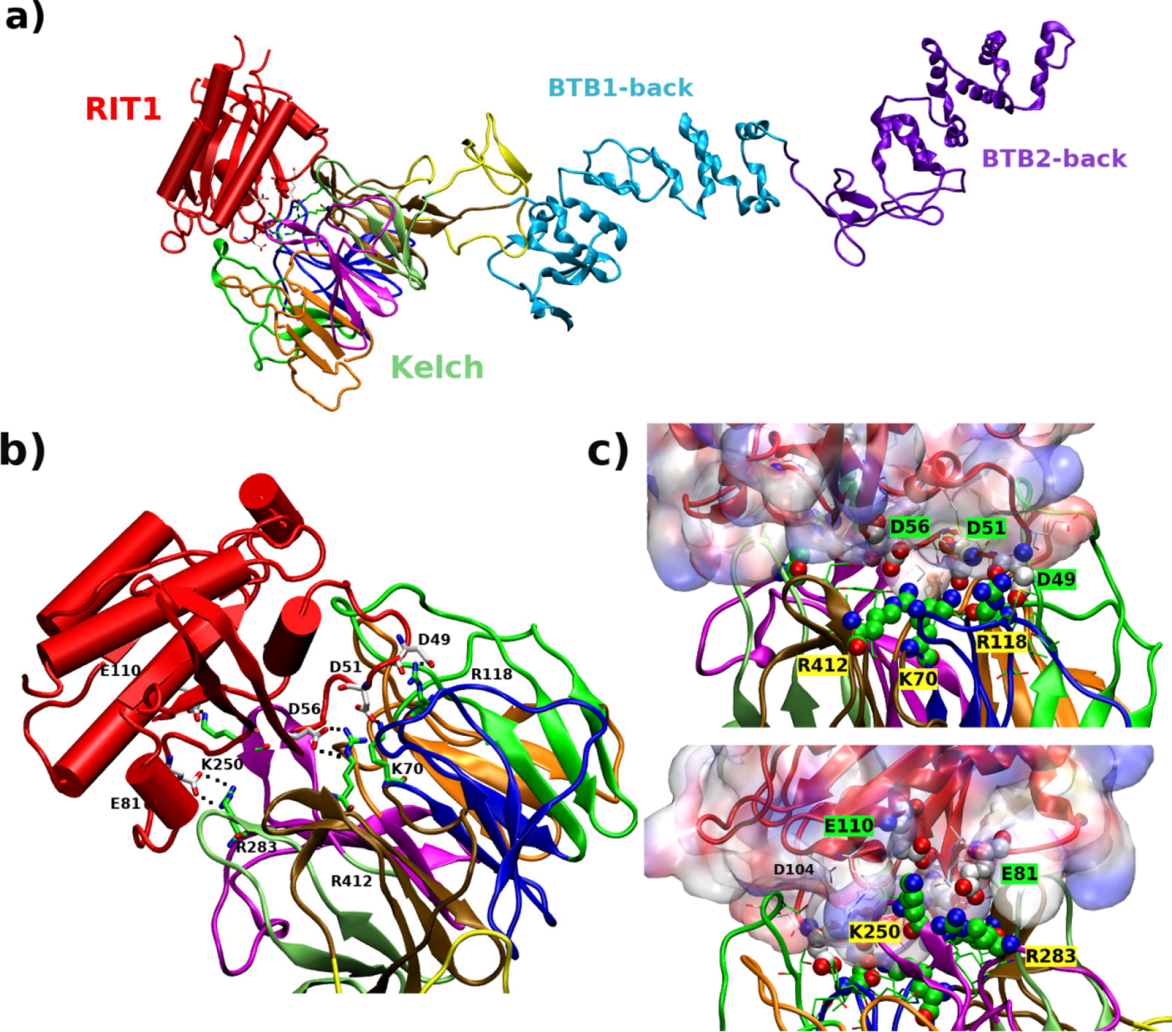
LZTR1-RIT1 docking model. (a) Docked RIT1 is rendered as red cartoons on the top of the Kelch domain. Structural motifs of LZTR1 are shown in cartoons and colored according to Figure 1. (b) Focus on the interaction between the LZTR1 Kelch domain and RIT1: bonded amino acids from LZTR1 and RIT1 are displayed as green and white sticks, respectively. (c) Zoom-in the protein–protein interface. Interacting amino acids are rendered in CPK, and ghost surface is used to display RIT1 in the same orientation as in (b) (upper panel) and its rotation of 180° around the *y* axis (bottom panel).

Given that, docking results have been selected when RIT1 and LZTR1 contact each other by way of those “mutationally” validated faces, which use point mutations as physical constraints (see Methods). Depth analysis of the top-ranked interaction modes provided a consensus binding pose for the LZTR1-RIT1 complex. Specifically, the selected LZTR1-RIT1 complex relies on binding interactions between K70, R118, R250, R283, and R412 from LZTR1 and D49, D51, D56, E81, and E110 from RIT1, as displayed in Figure 3. The selected model also displays the most favorable interaction energy as predicted by the FoldX energy function in an independent measure [first ranked complex ΔΔ*G* = −7.2; second complex ΔΔG = −5.3; third complex ΔΔ*G* = −0.70 kcal/mol].

Consistent with these results, the analysis of X-ray structures of some Kelch proteins in complex with small fragments of substrate molecules shows that the binding is mediated by a network (usually a pair of contacts from two different Kelch repeats) of electrostatic interactions between the positively charged amino acids of the Kelch and the negatively residues of the interacting partner. Structural comparison of the LZTR1-RIT1 complex to the Kelch domain of Keap 1 bound to the Nrf2 peptide (PDB code 2FLU) indicates an analogous and overlapping mode of interaction (see Figure S6).

### Mutational Scan of LZTR1: Differences between Free and Bound State

Depending on the spatial localization and chemical neighborhood, point mutations can affect biological activity at different stages, by modifying mechanical stability, structural features, and recognition patterns, eventually impacting on functional dynamics. Therefore, to validate our hypothesis on the LZTR1-RIT1 complex, we focused on mutational patterns of LTRZ1 and modeled and tested mutants of the interacting interface of LZTR1.

To this end, to identify the most interesting and impacting mutations of the Kelch domain, we applied a high-throughput saturation mutagenesis approach based on an effective empirical energy function implemented in FoldX to predict the effect on protein stability (ΔΔ*G*) induced by all the possible substitution of each germline mutation of LZTR1 reported in Table 1.^34^ Here, differences in folding energies are evaluated for both LZTR1 forms, in its free state and in complex with RIT1; position scanning analysis is carried out on the most representative conformations of the two protein states, obtained by MD simulations as discussed below.

**Figure 4:**
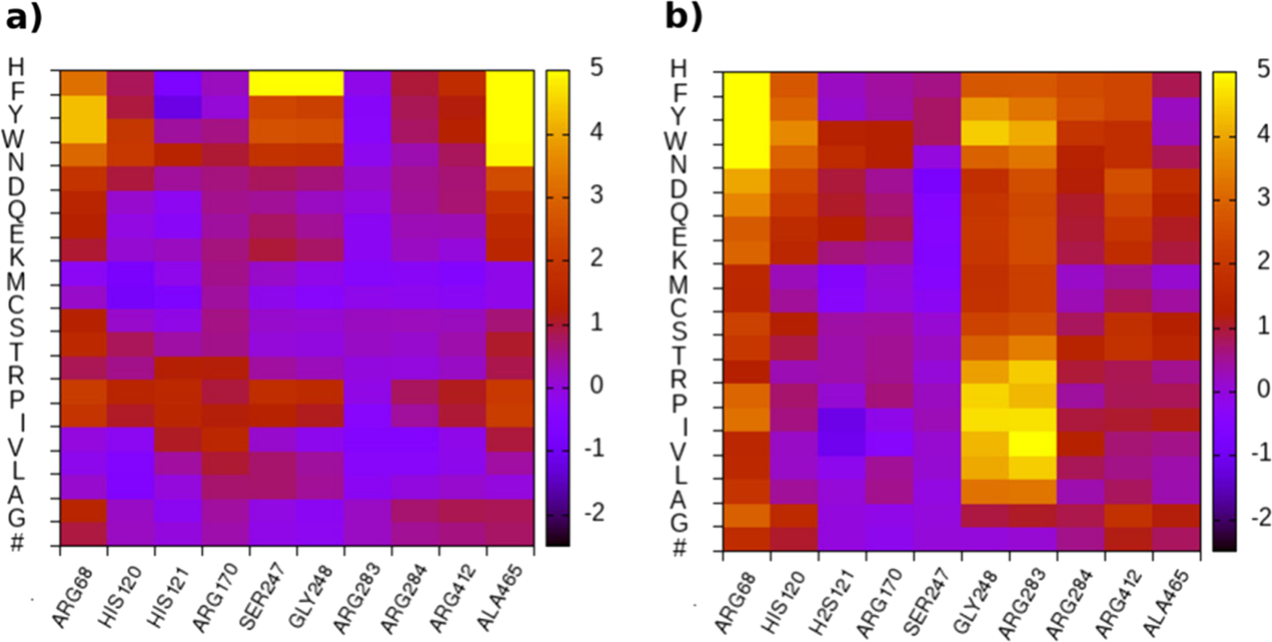
FoldX mutational analysis. Position scanning is applied to germline mutations annotated in Table 1 for free LZTR1 (a) and LZTR1 in complex with RIT1 (b). Calculations are run on the cluster centroids from MD simulations.

Changes in free energy of folding upon mutation in bound LZTR1 are displayed in Figure 4, where the largest deviation occurs at position 283. In other words, all replacements of arginine at 283 are associated to a significant destabilization of LZTR1 (ΔΔ*G* = Δ*G*_mut_ – Δ*G_wt_*) when bound to RIT1. Notice that this amino acid is also involved in an ionic interaction with RIT1 in the selected complex model (Figure 3), with the other annotated germline mutations R412C. The impact of mutations on protein stability and binding affinity is a key point to understand their functional effects*.* Besides protein stability, in fact, the substitution of single amino acids can alter the binding affinity to interactors when occurring at, or near, binding sites, or at distal sites through complex allosteric mechanisms. To this end, from the comparison of free energies of folding between the two systems, we selected mutations R283Q, R412C, responsible for direct interaction with RIT1, and G248R, localized at the upper face of LZTR1, not directly involved in the binding and that turns very destabilized when replaced by hydrophobic and bulkier residues (Figures 2d, 3, and 4).

On the other hand, mutagenesis scan carried out on RIT1 interaction sites showed that D49, D51, E55, D104, and E110 in particular are found to be sensitive to most substitutions (Figure S7). Further, the sequence alignment of several RAS proteins reveal an intriguing correlation with the different biochemical affinities reported so far, which requires dedicated investigation.^5,6,30,31^

### MD Assessment of the Effect of LZTR1 Mutations on Conformational Dynamics

**Scheme 1:**
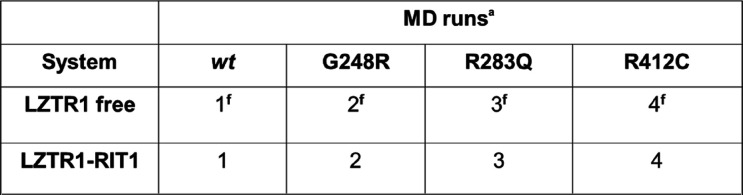
Set of MD Simulation Run on LZTR1 Two MD replicas per system are run and indicated with cardinal numbers I and II.

To assess the significance of our predictions on either intrinsic stability or recognition patterns, we run extensive molecular dynamics simulations of the full-length LZTR1 in its *wild-type* and mutated sequence. In our screening, G248R, R283Q (kelch-IV), and R412C (kelch-VI) are missense changes affecting highly conserved amino acids and predicted *in silico* to be destabilizing and damaging (highest pathogenicity score in Table S1; see Figures 2–3,4, Figure S5, and Table 1). Overall, we collected eight systems: *wt-*LZTR1 (denoted as **1** throughout the text), variants G248R-LZTR1 (denoted as **2**), R283Q-LZTR1 (denoted as **3**), and R412C-LZTR1 (denoted as **4**) were simulated both in the free form (superscript **f**) and in complex with RIT1, as summarized in Scheme 1.

Besides stability, conformational dynamics allows the simultaneous evaluation of the local effects caused by point mutations on the recognition surface of the two proteins and the characterization of the conformational response of LZTR1-RIT1 complex accompanying these residues substitutions.

### Wild-Type

**Figure 5:**
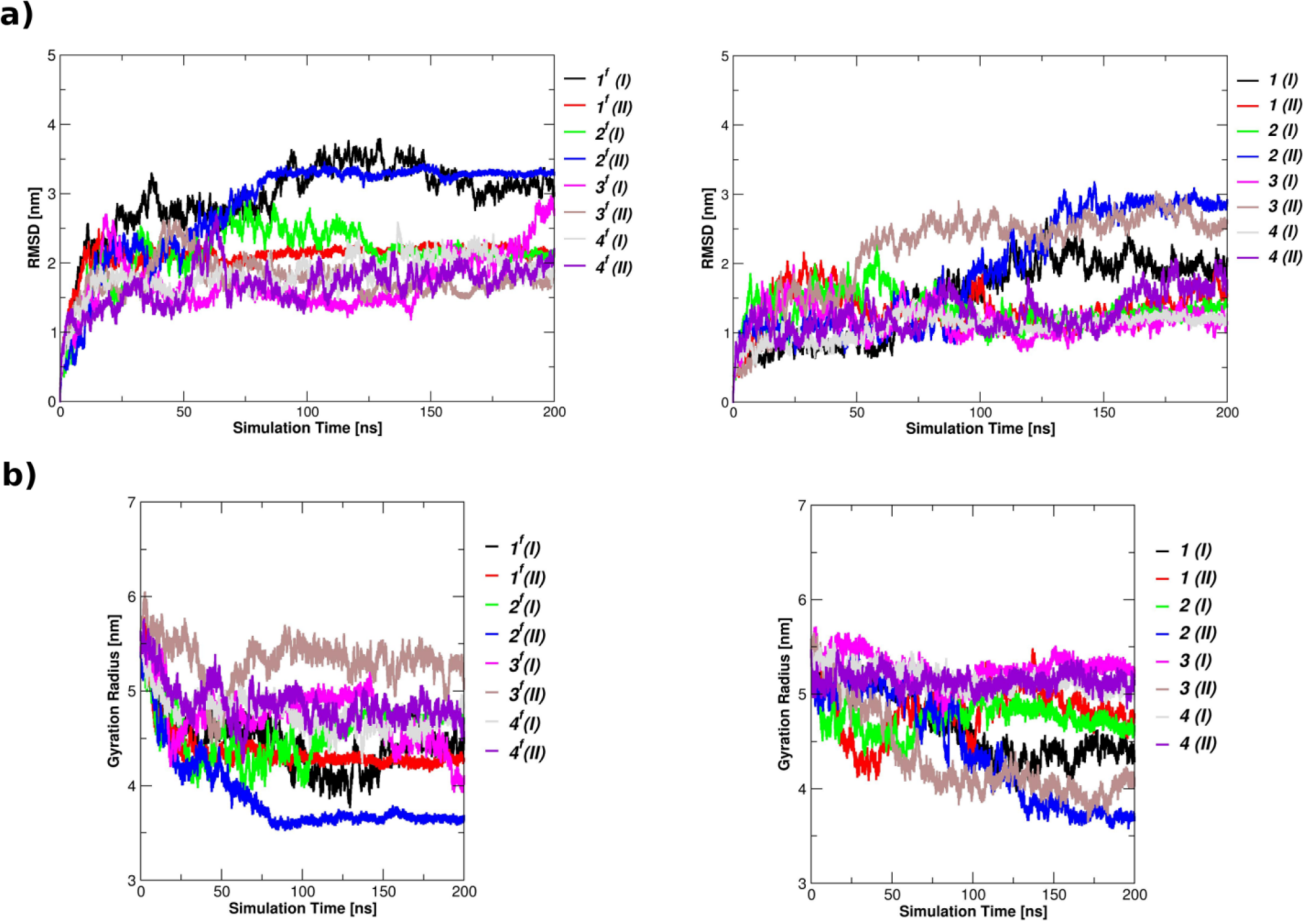
Root mean square deviation and gyration radius of LZTR1. RMSD (a) and Rg (b) are displayed for free LZTR1 (left) and bound LZTR1 (right) as a function of time. Cα atoms are used for calculations.

Along the MD simulation time, native LZTR1 shows significant and generalized structural fluctuations. Yet, Figure S3 indicates that such structural variations primarily concern interdomain adjustments as RMSD of single blocks (bottom panel in Figure S3b) drastically decrease as compared to atomic position deviations of the entire protein. The structural variability of *wt-*LZTR1 is a common feature of both systems **1^f^** and **1**, as confirmed by the evolution of both RMSD and gyration radius (Rg) (Figure 5). Nevertheless, it is also visible that binding of RIT1 reduces atomic position deviations and leads to an overall more compact LZTR1 form. In complex **1**, the degree of variability differs between replicas and, in the case of run 2, Rg stabilizes at a higher value as compared to run 1 (Figure 5). To this end, the analysis of the bond network at the interface between the two proteins shows that starting electrostatic interactions are fully (run 1) and partially (run 2) kept whereas the hydrogen bond number increases along both the simulations (Table 2 and Figures S8 and S9).

**Table 2:**
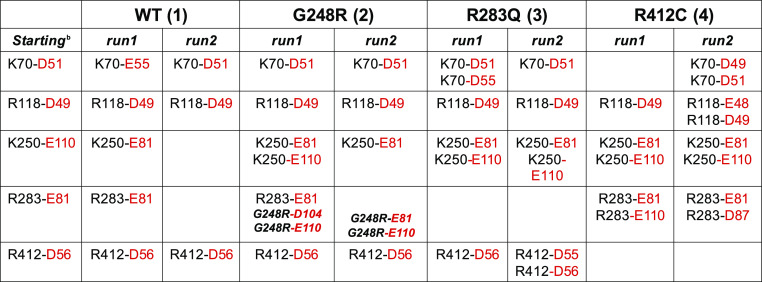
Salt Bridges in LZTR1-RIT1^a^

a RIT1 amino acids are in red. Bold labels are used for *ex novo* salt bridges in mutant G248R. See also Figure S9.

b After EM equilibration.

### G248R and R283Q Mutants

At a first glance, analyses of root mean square deviation and gyration radius confirm that all structures reach structural stability in the first nanoseconds of the simulation time (Figure 5). In particular, when bound to RIT1, the elongated LZTR1 goes through a very extensive atomic fluctuation, experiencing a significant structural stabilization and reshaping in a more compact form. The evaluation of Rg confirms that the differential spatial rearrangement is an intrinsic feature of LZTR1, uncorrelated with the mutation. Although the magnitude of the variation is different, unbound and bound LZTR1 share this behavior. In addition, the effect of LZTR1 plasticity does not impact the RIT1 binding, which minimally modifies its placement. At the binding interface, protein–protein interactions are further stabilized by the formation of new electrostatic bonds mediated by the G248R substitution in system **2** and recurring bidentate interactions of K250 and R412 from LZTR1 of both systems **2** and **3** (Table 2).

### R412C Mutant

In the case of R412C, structural fluctuations and local rearrangement seem to rely on the nature of the mutation as, either in the unbound or in the bound form, this mutant is associated to a minor re-organization (gray and violet lines in Figure 5). Interestingly, this is the only mutated system that exhibits a common behavior in all simulations, i.e., in the two types of systems and in all replicas, and LZTR1 undergoes a smaller change from the starting extended form regardless of whether it is free or complexed to RIT1. Therefore, molecular determinants of this structural stability should be searched into the LZTR1 sequence, and not at the protein–protein interaction, as observed in previous cases. In fact, in system **4**, missing R412-mediated interactions modify existing bond network, and in run 1, a smaller number of bonds holds RIT1 at the interface (Table 2 and Figure S8). The different conformational behavior of this mutant could depend on the exact position of the mutation (k-VI) and/or the identity of amino acid substitution R → C.

**Figure 6:**
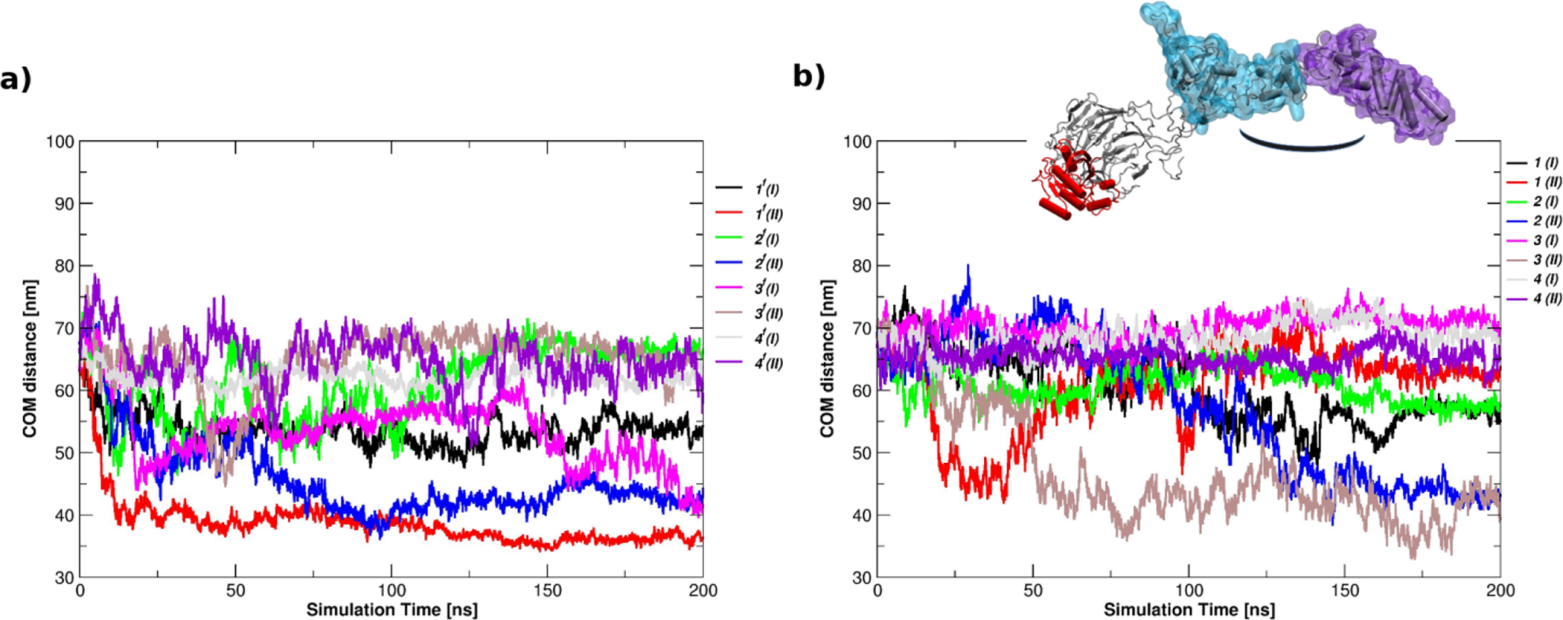
Center of mass distance. COM distances between the two BTB-Back domains in the free LZTR1 state (a) and in LZTR1-RIT1 complexes (b) are shown as a function of time.

The extended multi-domain conformation of LZTR1 itself boosts a high flexibility that can induce significant structural adjustments, as to minimize solvent exposure for instance (solvent accessible area decreases from 570 to ∼460 nm^2^ on average along the trajectories for system **1^f^**). Still, insights into the dynamical behavior show that R412C is associated with the lowest fluctuation rate spanned along the multi-domain structure, while large conformational mobility sampled by LZTR1 in the other systems mostly converges in the reciprocal movement of the two BTB domains. To this purpose, monitoring the evolution of center of mass distance (COM) along the simulation time between the two BTB-Back domains demonstrates large mutual fluctuation. It has been reported that BTB domains participate in the dimerization process and, in CRLs, provide the interacting region for Cullin 3. Therefore, the reciprocal rearrangement of these two blocks can play a crucial role in the ubiquitin ligase activity of the complex. Mutation at the Kelch domain is able to affect the global motility of the C-terminal domain. Figure 6 displays that R412C at the VI kelch repeat is associated to the most relevant effect on the BTB-Back domains displacement: while the two domains experience an extensive reciprocal fluctuation in the other bound and unbound systems (**1**, **1^f^**, **2**, **2^f^**, **3**, and **3^f^**), they appear more rigid and do not move at a comparable extent when R412C mutation is present, and RIT1 binding only marginally strengthens this behavior (**4** vs **4^f^**).

### Principal LZTR1 Conformers and Functional Implications

Next, considering differential conformational sampling, we asked whether the dynamical effect observed for R412C mutation could affect intra- and interdomain coordination and ultimately correlate with functional implications.

In particular, cluster analysis was applied to disclose mutation-dependent large amplitude motions and to identify the most representative conformations LZTR1 adopts along the simulation time, in complex with RIT1 and in its apo form.

**Figure 7:**
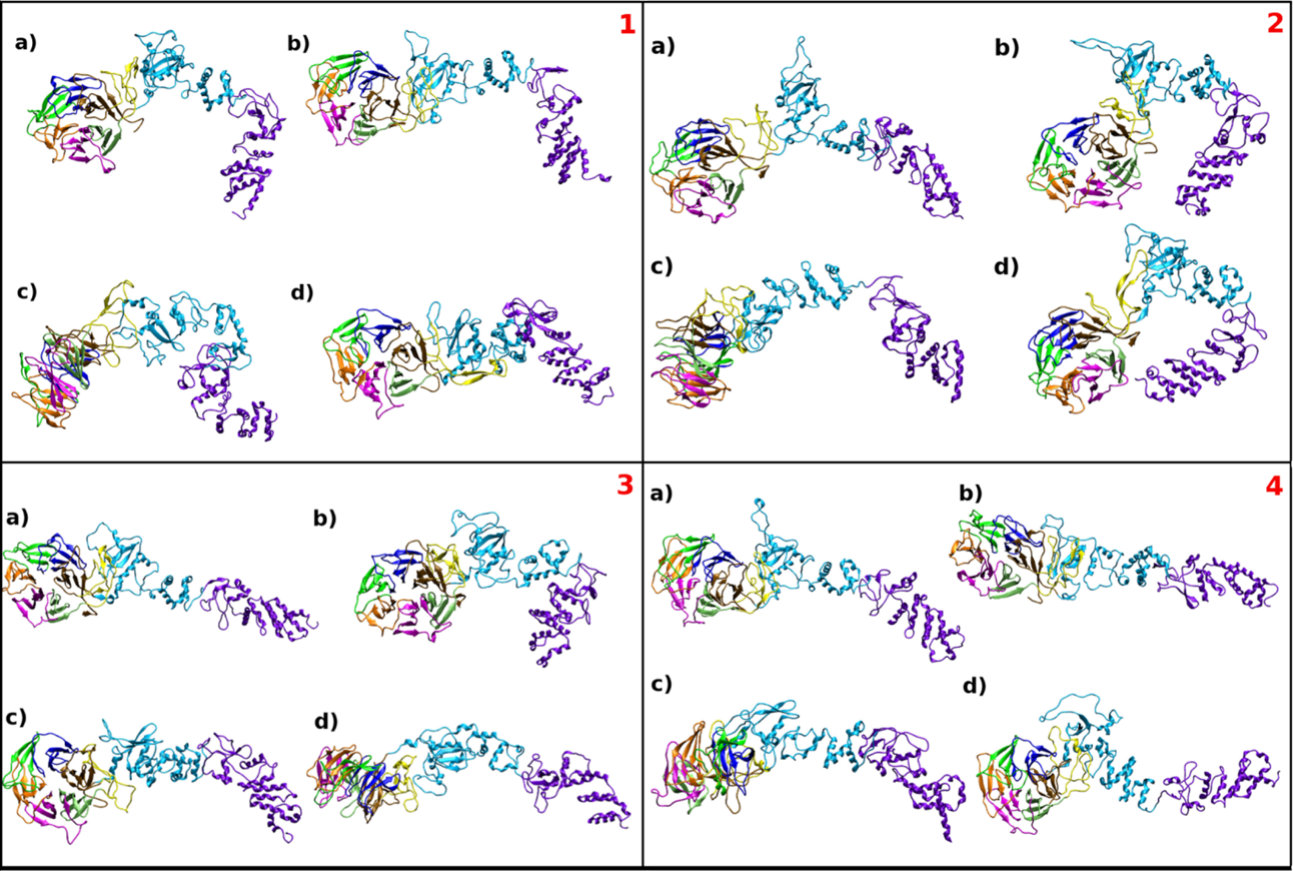
Cluster analysis. Structural representation of the first cluster per MD simulation: (1) *wild-type*, (2) G248R; (3) R283Q; (4) R412C. In each panel, (a, b) centroids of bound-LZTR1 in the two runs and (c, d) those of free-LZTR1. See Methods.

To produce comparable results, a cutoff of 1.0 was taken for all simulations as the average RMSD variation along all the trajectories (⟨rmsd⟩ = 1.0 nm) and centroids of the first cluster are displayed in Figure 7. The most superposable conformers belong to the simulation replicas of complex **4** (rmsd = 1.3 nm), while a larger deviation occurs, for example, between *wild-type* LZTR1 in its bound and free state (complexes **1** and **1^f^**, rmsd = 3.06 nm). In all systems, the representativeness of the first cluster centroid never goes below 54% of the cluster coverage (complex **2**), reaching up to 90% on average for R412C mutants. In other words, system **4** stabilizes an elongated conformer very similar to the starting structure for almost the entire trajectory.

How a small variation in a specific site is propagated along the structure to a distal point is a very debated question. To seek the onset of observed differential modulation, we looked at the spatial link between the N-term Kelch and the C-terminal BTB-Back domains, which is the disordered loop between k-V and k-VI repeats (Figure 1). Focusing on the local flexibility, from the evolution of root mean square fluctuations (RMSF) calculated as the time-average value per amino acid along MD trajectories, systems bearing R412C mutation are associated with the lowest fluctuations (Figure S10). We could reason that the presence of the SH group of cysteine at position 412, only minimally polar, affects the local rearrangement of kelch-VI increasing its hydrophobic packing and rigidifying the entire domain. As a consequence, due to its spatial proximity to the long disordered 325–385 loop together with the high motility of this linker, R412C substitution may eventually reverberate on the global dynamics of the following C-term BTB domains.^34^

### Changes in Protein–Protein Interactions Impact LZTR1 Conformational Sampling

Mutations on surface residues are more likely to interfere with the protein interaction rather than disrupting the protein fold; therefore, the mutation of a charged residue at the outer face of LZTR1 is expected to alter the molecular recognition of the two interacting partners. Indeed, the choice of R283 and R412 from the IV and VI blades, respectively, that form an ionic interaction with RIT1 negatively charged E81 and D56 is aimed at disrupting or, at least, disturbing LZTR1-RIT1 binding. Likewise, though unable to form direct bonds, the tiny G248 protrudes from the kelch-IV top face at the BC loop, motif known to provide the substrate-binding surface.^14^

Contrarily, we would not expect that the same variation will exert a visible effect on the global rearrangement of the unbound LZTR1.

With regard to LZTR1-RIT1 complexes, the detailed analysis of the interaction network made at the interface shows that starting electrostatic bonds between the two proteins are conserved along the trajectories (Figure 2). Interacting partners are kept in place by a combined cluster of electrostatic interactions and hydrogen bonds within the Kelch domain, specifically involving k-I (K70, R118), k-IV (R250, R283), and k-VI (R412) repeats (Table 2, Figure 3, and Figure S8). The presence of point mutations in the Kelch domain (R283Q and R412C) modulate this network as R283 (k-VI) and R412 (k-VI) directly mediate salt bridges with RIT1. Mutations induce a moderate local rearrangement of the binding mode: at the IV kelch repeat, substitution of arginine 283, on the DA loop, favors a stronger binding by K250 at the previous BC loop; whereas at the BC loop of the VI kelch repeat, mutation R412C promotes interactions at the I kelch level (run 2), involving K70 within the following DA loop. Notice that the replacement of glycine at 248 favors extra ionic bonds mediated by the arginine at the IV kelch level. The so-obtained bond network allows a stable recognition surface between LZTR1 and RIT1 and, depending on the amino acid replacement, only induces a small repositioning of RIT1.

It is worth noting that R283 and R412, which make two important contacts between LZTR1 and RIT1, are also involved in intramolecular salt bridges (R283-D304 and R412-D86) in the free-LZTR1 model and that these bonds can form at the same time (data not shown).

Moreover, the proposed protein–protein arrangement also agrees with the hydrophobic hub forming at one side of the interaction area that concurs to stabilize the binding: a π–π stacking between Y/H119-H120 and followed in sequence by H121 would rigidify a region predicted to play a crucial role in substrate binding activity.^29^

In this context, a tighter interaction between the two partners could modify (slow down) the turnover rate of the ligase ubiquitination process.

## Conclusions

In the present study, we modeled the three-dimensional structure of the full-length LZTR1 adaptor protein as a fully elongated multi-domain structure whereby the N-terminal Kelch domain appears well separated from the two following BTB-Back domains. While MD simulations highlighted large conformational dynamics, mainly concerning structural fluctuations of the disordered long loops and interdomain orientation, the Kelch domain does not experience significant structural changes along the simulation time and it is constantly available for substrate binding. These observations prompted us to investigate the interaction between LZTR1 and its target substrate and how this assembly reflects on protein motions.

The docked complex LZTR1-RIT1 provided additional evidences on the dynamical behavior of LZTR1 and helped to pinpoint key surface sites as top players in the recognition process. Experimental considerations (mutational data) have been used to guide our docking model contributing an improved picture of the recognition strategy used by E3 adaptor protein LZTR1 to bind RIT1. Our results on bond network at the LZTR1-RIT1 interface together with its intrinsic conformational variability agree with E3’s role in multiple target identification for efficient proteasomal degradation. Insights into differential dynamical response upon mutations aid to clarify the key role of pathogenic point mutations at the top face of the adaptor protein: while R283Q at the k-IV repeat modifies the interaction network at the LZTR1-RIT1 interface, an additional effect shows up when R412C is present at the k-VI repeat.

Because of its chemical–physical properties and its reactivity, cysteine is marginally exposed on protein surfaces; instead, buried free cysteine residue could more likely concern protein stability and folding. In our simulations, this mutant is associated with reduced conformational variability, suggesting a contribution not limited to the recognition mechanism, hence shedding new light on the mechanistic role of several missense alterations localized in BTB-Back domains and already discussed.^1,3,5,31^

Understanding the molecular basis that underpin the functional dynamics of proteins and guide the recruitment and the interaction between different molecular partners is fundamental for the definition of the molecular mechanisms underlying diseases and drug development processes.

E3 ligases catalyze ubiquitin transfer and subsequent proteasomal degradation of specific substrates, thus controlling a plethora of biological processes. A better understanding of E3-substrate network may yield fundamental opportunities for drug development (structure-guided ligand design). Today, there are hundreds of putative E3 ligases, but many are scarcely characterized, especially considering individual protein substrates.^35^ Overall, our simulations show that LZTR1, both in the free and complexed states, experience large structural movements and interdomain fluctuations. As a matter of fact, no experimental 3D structure for LZTR1 is available to date and the three juxtaposed domains (Kelch-BTB1/Back-BTB2/Back) adopt an elongated and highly dynamic shape. To our opinion, this feature may add an extra pitfall for the identification of E3 ligase substrates, besides the dynamic nature of protein ubiquitination, the transient interactions between ligase and substrate, the multiplicity/redundancy of substrates targeted by E3 ligases.^35^

In this context, our interaction model represents the first attempt of structural and dynamical characterization of the interaction between E3-ligase binding protein LZTR1 and its substrate RIT1, with an eye on functional implications.

Interestingly, the modular architecture of the single-chain adaptor protein may share similar functionalities already reported for other CRL multimeric complexes, whereby coupled motions among distal elements are thought to allosterically regulate substrate ubiquitination.^22,24,26,36^

It is known that molecular structure and dynamics are at the basis of the biological function and that the perturbation of this delicate relationship is responsible for incorrect protein activity. Molecular recognition, binding phenomena, enzyme catalysis strongly rely on coupled conformational motions, and even small single changes are capable to induce large structural and functional switches. In this context, fine functional regulation can be achieved in the cell by allosteric mechanism by which an event/perturbation at a certain site can tune enzymatic activity or binding affinity in a distal region, leading to the activation of specific conformational states that meet functional requirements.^37−40^

Including protein flexibility and dynamics can boost the characterization of biomolecular pathways, and in particular, it has been shown that CRL flexibility can play a key role in ubiquitination.^25,41^ Liu et al. underscored the significance of multi-complex dynamics in controlling the E3 activation process, facilitating the ubiquitin transfer reactions.^41^ By molecular dynamics simulations, they demonstrated that several cullins (Cul1, Cul4A, and Cul5) and nine different substrate-binding proteins bound to E3 elements display a diverse degree of structural flexibility: components of the multi-domain complex emerge as flexible scaffolds, whereby allostery-driven E3 CRL activation is coupled to the conformational change mediated by cooperative/concerted modulation of spatially distal sites.^21−23^

**Figure 8:**
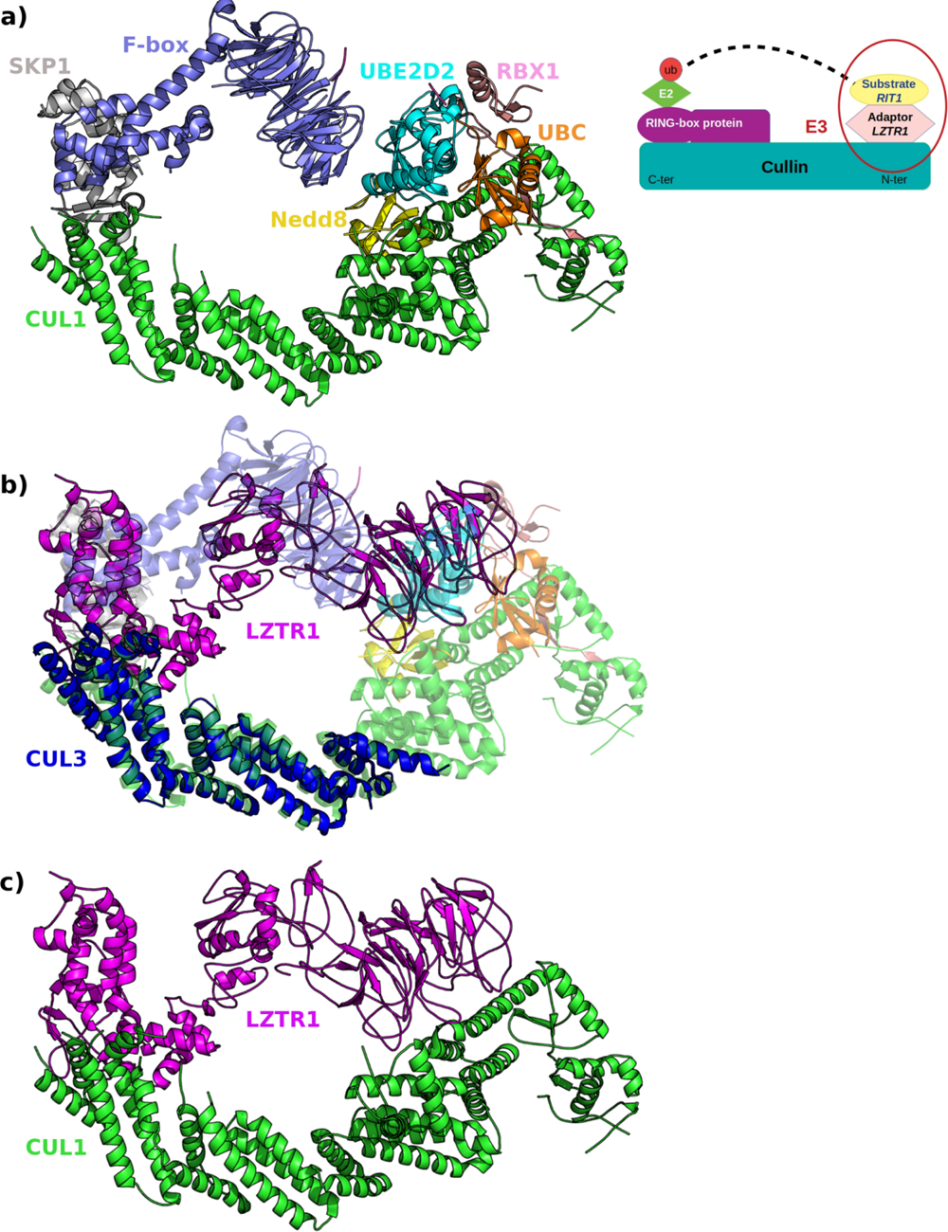
LZTR1-Cul3 dynamics. Reconstruction of LZTR1-Cul3 complex. (a) Cryo-EM structure of the CRL1β-TRCP–UBE2D∼Ub–IkBα assembly (PDB 6TTU): Cul1 and adaptor Skp1 are rendered in green and violet cartoons, respectively. (b) Complex between the LZTR1 (magenta) conformer along MD simulations (system 2) and Cul3 (blue) (from the superposition on the X-ray structure of the SPOP BTB domain complexed with the Cul3 N-terminal domain, PDB 4EOZ) superposed on the correspondent adaptor and cullin of the cryo-EM assembly. (c) Putative complex between the representative LZTR1 conformation and full-length Cul1. Inset: Schematic representation of the Cullin RING-E3 ligases assembly.

Furthermore, recent cryo-EM data provide a comprehensive mechanistic explanation for the full model of ASB9-CRL assembly, focusing on conformational dynamics that underlie substrate recognition and ubiquitin loading.^26^ Also, a late research by the Schulman group reports the cryo-EM structure of the multivalent cullin-RING-UBE2D ubiquitin ligation assembly. The authors disclose the conformational dynamics at the basis of ubiquitin transfer mediated by the neddylated CRL1β-TRCP from UBE2D to the substrate IkBα.^27^ In the proposed mechanistic model, conformational changes required to reach the catalytic architecture could be achieved through several ways and flexibility can concern different players of the assembly. In this scenario, our molecular dynamics studies highlight the capability of the adaptor protein to sample different conformations. We morph the LZTR1-Cul3 complex using the crystal structure of the SPOP BTB domain complexed with the Cul3 N-terminal domain (PDB 4EOZ)^42^ and then superimpose the correspondent biological partners of the CRL1β-TRCP–UBE2D∼Ub–substrate assembly (Cul3 on Cul1; LZTR1 on Skp1). Subsequently, with a more compact LZTR1 conformer (i.e., cluster centroid of system 2, Figure 7,2b), we obtain an interaction model with structural relationships LZTR1/Cul3 that resemble those observed in functional CRL1β-TRCP–UBE2D∼Ub–IkBα. In particular, the large mobility of the two BTB-Back domains facilitates comparable distancing of their counterparts in the catalytic assembly. Figure 8 informs on the large plasticity shown by LZTR1, which may accomplish an accurately positioning and orienting of the substrates for ubiquitin transfer in the correspondent CRL3 machine. Each component of the CRLs can play a big part in the finely regulated functional dynamics, thus reconciling with the emerging view of the allosteric control of ubiquitin ligase activity.

Of interest, several experimentally determined BTB domains have proven able to mediate dimerization and various modes of self-association. Higher-order oligomerization could act as a catalyst for the efficiency and versatility of substrate ubiquitination.^10,11,42^ Large amplitude mobility of the LZTR1 adaptor observed in molecular dynamics simulations may support the possibility for BTB-Back domains to self-interact in an intradomain dimerization mechanism as well, carrying delicate implications on Cul3 interaction. However, this aspect goes beyond the scope of the present study and will be addressed in future work. Furthermore, our research allowed us to reveal a differential dynamical effect driven by mutations, define surface subregions in Kelch repeats primarily involved in target recognition process, and identify key mutations to be tested experimentally. The nature of the electrostatic interactions between the adaptor (positive) and the substrate (negative) opens to the design of negatively charged compounds to optimize electrostatic complementarity and assess LZTR1 recruitment properties.^43^

Although structural and functional implications discussed above rely on important assumptions, modular architecture and long flexible loops of LZTR1 already holds a functional dynamics role: we speculate that flexibility at this level could be a distinctive feature of Cul3-based ligases as adaptor and substrate-receptor functions are merged into a single polypeptide, therefore providing additional evidences that the mechanism for ubiquitination is under conformational control.

We believe that our findings help putting one more brick into the understanding of E3-substrate network and in the definition of the pharmacophoric requirements for the development of PPI targeting molecules with therapeutic potential.

## Methods

### Homology Modeling

Homology models of LZTR1 were built using MODELLER software (v 9.23).^44^

HHPred^45^ was employed for homology detection of structure templates. HHPred predictions identified the crystal structure of the Kelch domain of the thiocyanate-forming protein from *Thlaspi arvense* (PDB code 5A10) for the Kelch domain (score 268.9) and the X-ray structure of the human SPOP-BTB domain (PDB code 4J8Z) (score 174.9) for the BTB domains. These pairwise query-template alignments are used to generate the 3D structure using MODELLER.

We built a multi-template homology model of the full-length LZTR1 sequence (UniProtKB: Q8N653) by modeling individually the Kelch and the two BTB1-Back motifs and joining the three domains in order to reduce approximations due to the large disordered loops (∼aa 420–430, ∼aa 632–660) and alignment score (limited sequence similarity) (see Figure S1). The MODELLER scoring function scored the best modeled structures using the discrete optimized protein energy (DOPE) method.^46^ In this ranking, the Kelch domain was assigned −35498.96 kcal/mol; BTB1-Back scored −19588.38 kcal/mol; BTB2-Back −18385.77 kcal/mol.

- Kelch-5a10 (1-423 lztr1) (1-460 5a10) = 24.3% sequence identity; 30.5% similarity;
- BTB1-4J8Z (421-631 lztr1) (178-356 4J8Z) = 26.9% sequence identity; 37% similarity;
- BTB2-4J8Z (661-828 lztr1) (194-356 4J8Z) = 28.8% sequence identity; 42% similarity.

To further support structural modeling and to cover non-aligned regions, Jpred predictor^47^ was used for secondary structure predictions for the full-length LZTR1 sequence (see Figure S2).

It is worth noting that the loop linking (∼aa 630–652) between the two BTB-Back domains was added and automatically refined using the Maestro suite (Schrödinger release 2019-4, LLC, New York, NY) and does not reflect a proven orientation of the two motifs; in this context, MD simulations are meant to explore larger conformational rearrangements.

MolProbity^48^ was used to estimate the quality of the obtained LZTR1 structural model. Evaluation of homology models suggest a high degree of reliability (with 93% of residues in favored and allowed regions of the Ramachandran plot) except for some distorted geometry found at the poorly structured loops connecting the three domains (∼ 420 and ∼630).

### Pathogenicity Prediction

We collected known missense LZTR1 somatic and germline mutations from available databases COSMIC (https://cancer.sanger.ac.uk/cosmic) and The Cancer Genome Atlas (https://www.cancer.gov/tcga).

The prediction of pathogenicity was carried out by using four different predictors, SIFT,^49^ Polyphen-2,^50^ MutationTaster2,^51^ and Provean-Pred:^52^ mutation is pathogenic when analyzed alterations are scored as damaging by the four predictors.^53^

The full list of 252 missense variations covering the full-length LZTR1 sequence is given in the Supporting Information (Table S1).

To evaluate the variant origin and the clinical significance of disease-associated LZTR1 germline mutations, we retrieved data from the ClinVar public archive (https://www.ncbi.nlm.nih.gov/clinvar/).

FoldX (http://foldxsuite.crg.eu) predicts changes in free energy of folding upon mutation as the difference between the estimated free energy of folding of the mutant and the reference *wild-type* variants. The empirical free energy function includes terms for van der Waals interactions, solvation free energies, water bridges, hydrogen bonding, electrostatics, and entropy changes upon folding for main chains and side chains. Moreover, to improve ΔΔ*G* estimations, free energy predictions were calculated on conformers from molecular dynamics simulations.^34,54^

### Docking Model

ClusPro,^55^ Zdock,^56^ PatchDock,^57^ and Frodock^58^ software was used to build the LZTR1-RIT1 interaction model.

We run both blind docking experiments, which are performed with no structural indication about the interacting surfaces, and guided docking experiments (Zdock), which are carried out by explicitly selecting binding sites on the RIT1 surface (A57, A77, E81, F82, T83, Y89, and M90). Moreover, due to size limitations for the docking calculation (ClusPro), we also run docking runs where the BTB2-Back domain at the C-terminal was removed. Docking results were thus screened to select a consensus interaction mode between the two proteins, with the most favorable energy score and consistent with recent experimental indications on RIT1 at the same time.^6^ To reduce structural inaccuracies due to the modeling procedure, docking was performed using a representative MD structure of LZTR1 and RIT1 (after structural refinement and 10 ns of MD) as starting structures.

### MD Simulations Setup

Molecular dynamics simulations were performed using Gromacs v. 5.1.5^59^ by applying the Amber03^60^ force field. The structures were centered in triclinic boxes at a 0.9 nm distance from each box edge and solvated with TIP3P^61^ water molecules; counter-ions were randomly added to ensure the overall charge neutrality, and periodic conditions were applied in all three dimensions. Bond lengths involving hydrogens were restrained using the LINCS algorithm.^62^ Electrostatic interactions were treated using the particle mesh Ewald method.^63^ Each system was first energy minimized using the steepest-descent method. Next, all systems were subjected to a 100 ps equilibration phase in the NVT ensemble: temperature coupling was kept by the velocity rescale thermostat scheme^64^ followed by an additional 100 ps equilibration step in the NPT ensemble, reached by coupling pressure with a Parrinello–Rahman barostat.^65^

After equilibration, MD production runs of 200 ns per system were carried out in an NPT ensemble (*T* = 300 K, *P* = 1.0 bar). To enhance sampling, two independent replicas with different initial velocities were run for each system: LZTR1-RIT1 complexes **1** (*wt*)*,***2** (G248R), **3** (R283Q), **4** (R412C); free LZTR1 (**1^f^**, **2^f^**, **3^f^**, and **4^f^**).

#### Interactions Analysis

Salt bridges along the trajectories are calculated by the VMD^66^ routine using a 3.2 Å oxygen–nitrogen distance cutoff. H-bonds are considered if hydrogen donor-acceptor angles are <30° and donor–acceptor distances are <3.5 Å by Gromacs utilities.

#### Center of Mass (COM) Distances Analysis

Distance length evolution between the two BTB-Back domains centroids were analyzed by the VMD tool package. Centroids were defined as the center of mass of BTB1-Back (amino acids 429–631) and BTB2-Back domains (amino acids 651–833). The distance between the two obtained geometric centers was calculated along the simulation time for complexes **1**, **2**, **3**, and **4** and for the unbound LZTR1 **1^f^**, **2^f^**, **3^f^**, and **4^f^** every 100 ps.

#### Cluster Analysis

Clustering was performed on LZTR1 in complex and in the unbound form, fitting on Cα atoms, using the gromos method^67^ by superimposing Cα atoms along MD trajectories and setting the RMSD cutoff at 1.0 nm. The centroid of the most populated cluster is given as the most representative structure of the conformational ensemble.

### Data and Software Availability

Genetic missense mutations occurring in LZTR1 have been annotated using the AnnoVar^53^ algorithm, which aggregates information from genomic and protein resources with cancer and non-cancer variant databases. The functional effect of the missense mutations on the LZTR1 protein was determined according to the damaging score calculated with AnnoVar through four prediction algorithms: SIFT,^49^ Polyphen2,^50^ MutationTaster2,^51^ and Provean.^52^ Nucleotide variants predicted as damaging by two or more algorithms were classified as pathogenic mutations. Authors will release mutational data along with the atomic coordinates of the produced models of the full-length LZTR1 and RIT1-LZTR1 complex upon article publication using the Zenodo.org repository.

## Supporting Information Available

(Table S1) Lztr1 mutational collection from Cosmic and TCGA databases; (Figure S1) pairwise sequence alignment used for the homology modeling of the human leucine zipper-like transcriptional regulator 1 (LZTR1); (Figure S2) Jpred Secondary Structure predictions for the human leucine zipper-like transcriptional regulator 1 (LZTR1); (Figure S3) LZTR1 structural stability, secondary structure and root mean square deviation for the two LZTR1 MD runs; (Figure S4) 3D structure of the RIT1 protein (PDB code 4KLZ); (Figure S5) pathogenic mutations found in human Lztr1 gene in Schwannomatosis, Noonan syndrome, and glioblastoma disorders; (Figure S6) structure superposition of substrate-binding proteins; (Figure S7) FoldX mutational analysis of RIT1 interacting sites; (Figure S8) hydrogen bonds made between LZTR1 and RIT1; (Figure S9) focus on protein–protein interaction of the *wild-type* complex from MD simulations; and (Figure S10) loop RMSF, average root mean square fluctuation per 325–385 loop residue (PDF)
