# Supplementary material for: Structural Model for Recruitment of RIT1 to the LZTR1 E3 Ligase: Evidences from an Integrated Computational Approach
Source: J Chem Inf Model. 2021 Apr 1;61(4):1875–88. doi: 10.1021/acs.jcim.1c00296 (PMC8154269; doi:10.1021/acs.jcim.1c00296)
Supplement: Supplementary file 1 — ci1c00296_si_001.pdf [file ci1c00296_si_001.pdf]

**Supporting information**

# Structural model for recruitment of RIT1 to the LZTR1 E3 ligase: evidences from an integrated computational approach

Antonella Paladino[1*], Fulvio D'Angelo[1,2], Teresa Maria Rosaria Noviello[1,3], Antonio Iavarone[2,4,5], Michele Ceccarelli [1,3*]

[1]BIOGEM Istituto di Ricerche Genetiche "G. Salvatore", via Camporeale, 83031 Ariano Irpino, Italy
[2] Institute for Cancer Genetics, Columbia University, 1130 St Nicholas Ave, New York, NY 10032, USA
[3] Department of Electrical Engineering and Information Technology (DIETI), University of Naples "Federico II", Via Claudio 21, 80128 Naples, Italy
[4] Department of Pathology and Cell Biology, Columbia University Medical Center, 1130 St Nicholas Ave, New York , NY 10032 USA
[5] Department of Neurology, Columbia University Medical Center, 1130 St Nicholas Ave, New York, NY 10032, USA
*Authors to whom correspondence should be addressed: antonella.paladino@biogem.it; michele.ceccarelli@unina.it

**Table S1. LZTR1 mutational collection from Cosmic and TCGA databases**. Pathogenicity is determined from independent predictions by SIFT[50], Polyphen[51], Taster-Pred[52], Provean-Pred[53] and it is assigned when mutation is predicted to be damaging by ≥ 2 predictors. Mutations distribution along LZTR1 sequence is shown in Figure S5.

| Assembly | Chr | HGVSc | HGVSp | Position | Pathogenicity | Damaging |
|---|---|---|---|---|---|---|
| GRCh37 | 22 | c.50C>T | p.A17V | 17 | tolerated | 1 |
| GRCh37 | 22 | 51_52delinsC | p.G18S | 18 | pathogenic | 2 |
| GRCh37 | 22 | c.55G>T | p.G19C | 19 | pathogenic | 2 |
| GRCh37 | 22 | c.61C>T | p.R21W | 21 | pathogenic | 3 |
| GRCh37 | 22 | c.62G>T | p.R21L | 21 | pathogenic | 3 |
| GRCh37 | 22 | c.76C>T | p.P26S | 26 | tolerated | 1 |
| GRCh37 | 22 | c.90C>G | p.F30L | 30 | pathogenic | 3 |
| GRCh37 | 22 | c.106G>T | p.D36Y | 36 | pathogenic | 2 |
| GRCh37 | 22 | c.122T>C | p.L41P | 41 | pathogenic | 4 |
| GRCh37 | 22 | c.154C>A | p.H52N | 52 | pathogenic | 3 |
| GRCh37 | 22 | c.155A>T | p.H52L | 52 | pathogenic | 3 |
| GRCh37 | 22 | c.157C>T | p.R53C | 53 | pathogenic | 4 |
| GRCh37 | 22 | c.160T>A | p.W54R | 54 | pathogenic | 4 |
| GRCh37 | 22 | c.163C>T | p.R55W | 55 | pathogenic | 4 |
| GRCh37 | 22 | c.166C>T | p.R56C | 56 | pathogenic | 4 |
| GRCh37 | 22 | c.179G>C | p.C60S | 60 | pathogenic | 4 |
| GRCh37 | 22 | c.183C>A | p.D61E | 61 | tolerated | 1 |
| GRCh37 | 22 | c.187T>C | p.F63L | 63 | pathogenic | 4 |
| GRCh37 | 22 | c.202C>G | p.R68G | 68 | pathogenic | 4 |
| GRCh37 | 22 | c.202C>T | p.R68C | 68 | pathogenic | 4 |
| GRCh37 | 22 | c.203G>A | p.R68H | 68 | pathogenic | 4 |
| GRCh37 | 22 | c.202C>A | p.R68S | 68 | pathogenic | 4 |
| GRCh37 | 22 | c.220G>A | p.V74M | 74 | pathogenic | 3 |
| GRCh37 | 22 | c.226T>A | p.Y76N | 76 | pathogenic | 4 |
| GRCh37 | 22 | c.248T>C | p.F83S | 83 | pathogenic | 4 |
| GRCh37 | 22 | c.251G>T | p.G84V | 84 | pathogenic | 4 |
| GRCh37 | 22 | c.256G>T | p.D86Y | 86 | pathogenic | 3 |
| GRCh37 | 22 | c.259A>G | p.N87D | 87 | pathogenic | 2 |
| GRCh37 | 22 | c.263G>T | p.G88V | 88 | pathogenic | 4 |
| GRCh37 | 22 | c.267G>C | p.K89N | 89 | pathogenic | 3 |
| GRCh37 | 22 | c.273G>C | p.M91I | 91 | pathogenic | 4 |
| GRCh37 | 22 | c.289C>T | p.R97W | 97 | pathogenic | 4 |
| GRCh37 | 22 | c.290G>A | p.R97Q | 97 | pathogenic | 4 |
| GRCh37 | 22 | c.295G>A | p.D99N | 99 | pathogenic | 3 |
| GRCh37 | 22 | c.311C>G | p.S104C | 104 | pathogenic | 4 |
| GRCh37 | 22 | c.311C>T | p.S104F | 104 | pathogenic | 4 |
| GRCh37 | 22 | c.313T>C | p.W105R | 105 | pathogenic | 4 |
| GRCh37 | 22 | c.314G>T | p.W105L | 105 | pathogenic | 4 |
| GRCh37 | 22 | c.319A>T | p.R107W | 107 | pathogenic | 4 |
| GRCh37 | 22 | c.320G>A | p.R107K | 107 | pathogenic | 2 |
| GRCh37 | 22 | c.322G>A | p.A108T | 108 | pathogenic | 2 |
| GRCh37 | 22 | c.338C>T | p.T113I | 113 | pathogenic | 2 |

| | | | | | | |
|---|---|---|---|---|---|---|
| GRCh37 | 22 | c.344C>T | p.P115L | 115 | pathogenic | 4 |
| GRCh37 | 22 | 347_348delins | p.A116V | 116 | pathogenic | 4 |
| GRCh37 | 22 | c.353G>A | p.R118H | 118 | pathogenic | 4 |
| GRCh37 | 22 | c.352C>T | p.R118C | 118 | pathogenic | 4 |
| GRCh37 | 22 | c.361C>G | p.H121D | 121 | pathogenic | 4 |
| GRCh37 | 22 | c.362A>G | p.H121R | 121 | pathogenic | 4 |
| GRCh37 | 22 | c.365C>T | p.S122L | 122 | pathogenic | 4 |
| GRCh37 | 22 | c.379G>T | p.G127W | 127 | pathogenic | 4 |
| GRCh37 | 22 | c.399T>A | p.F133L | 133 | pathogenic | 3 |
| GRCh37 | 22 | c.401G>A | p.G134E | 134 | pathogenic | 4 |
| GRCh37 | 22 | c.403G>A | p.G135S | 135 | pathogenic | 4 |
| GRCh37 | 22 | c.410C>T | p.T137I | 137 | pathogenic | 4 |
| GRCh37 | 22 | c.416A>C | p.D139A | 139 | pathogenic | 4 |
| GRCh37 | 22 | c.418A>G | p.I140V | 140 | pathogenic | 3 |
| GRCh37 | 22 | c.428A>C | p.N143T | 143 | pathogenic | 4 |
| GRCh37 | 22 | c.454C>G | p.L152V | 152 | pathogenic | 3 |
| GRCh37 | 22 | c.479G>A | p.G160D | 160 | pathogenic | 4 |
| GRCh37 | 22 | c.488C>T | p.T163M | 163 | pathogenic | 2 |
| GRCh37 | 22 | c.508C>T | p.R170W | 170 | pathogenic | 4 |
| GRCh37 | 22 | c.509G>A | p.R170Q | 170 | pathogenic | 2 |
| GRCh37 | 22 | c.518T>C | p.V173A | 173 | tolerated | 1 |
| GRCh37 | 22 | c.542C>T | p.T181M | 181 | pathogenic | 3 |
| GRCh37 | 22 | c.551G>T | p.S184I | 184 | pathogenic | 4 |
| GRCh37 | 22 | c.554A>G | p.D185G | 185 | tolerated | 1 |
| GRCh37 | 22 | c.555C>G | p.D185E | 185 | tolerated | 1 |
| GRCh37 | 22 | c.565A>T | p.I189F | 189 | pathogenic | 4 |
| GRCh37 | 22 | c.580G>A | p.D194N | 194 | pathogenic | 4 |
| GRCh37 | 22 | c.583G>A | p.G195S | 195 | pathogenic | 4 |
| GRCh37 | 22 | c.583G>T | p.G195C | 195 | pathogenic | 4 |
| GRCh37 | 22 | c.586A>T | p.N196Y | 196 | pathogenic | 4 |
| GRCh37 | 22 | c.587A>T | p.N196I | 196 | pathogenic | 4 |
| GRCh37 | 22 | c.589G>T | p.A197S | 197 | pathogenic | 2 |
| GRCh37 | 22 | c.590C>T | p.A197V | 197 | pathogenic | 3 |
| GRCh37 | 22 | c.592A>G | p.R198G | 198 | pathogenic | 4 |
| GRCh37 | 22 | c.599A>T | p.N200I | 200 | pathogenic | 4 |
| GRCh37 | 22 | c.600T>A | p.N200K | 200 | pathogenic | 4 |
| GRCh37 | 22 | c.602A>G | p.D201G | 201 | pathogenic | 4 |
| GRCh37 | 22 | c.614T>G | p.I205S | 205 | pathogenic | 3 |
| GRCh37 | 22 | c.644G>T | p.W215L | 215 | pathogenic | 4 |
| GRCh37 | 22 | c.674C>T | p.P225L | 225 | pathogenic | 4 |
| GRCh37 | 22 | c.677C>T | p.P226L | 226 | pathogenic | 4 |
| GRCh37 | 22 | c.693C>A | p.F231L | 231 | pathogenic | 4 |
| GRCh37 | 22 | c.719T>A | p.M240K | 240 | pathogenic | 4 |

| | | | | | | |
|---|---|---|---|---|---|---|
| GRCh37 | 22 | c.725T>A | p.V242E | 242 | pathogenic | 4 |
| GRCh37 | 22 | c.729C>A | p.F243L | 243 | pathogenic | 3 |
| GRCh37 | 22 | c.737A>G | p.Q246R | 246 | pathogenic | 4 |
| GRCh37 | 22 | c.740G>A | p.S247N | 246 | pathogenic | 3 |
| GRCh37 | 22 | c.742G>A | p.G248R | 248 | pathogenic | 4 |
| GRCh37 | 22 | c.752T>G | p.I251R | 251 | pathogenic | 4 |
| GRCh37 | 22 | c.755C>T | p.T252I | 252 | pathogenic | 4 |
| GRCh37 | 22 | c.758A>G | p.N253S | 253 | pathogenic | 3 |
| GRCh37 | 22 | c.763C>A | p.L255I | 255 | pathogenic | 3 |
| GRCh37 | 22 | c.763C>T | p.L255F | 255 | pathogenic | 4 |
| GRCh37 | 22 | c.770A>G | p.Q257R | 257 | pathogenic | 3 |
| GRCh37 | 22 | c.800G>A | p.R267H | 267 | pathogenic | 4 |
| GRCh37 | 22 | c.815A>G | p.H272R | 272 | pathogenic | 3 |
| GRCh37 | 22 | c.826G>T | p.G276C | 276 | pathogenic | 4 |
| GRCh37 | 22 | c.830C>T | p.S277F | 277 | pathogenic | 4 |
| GRCh37 | 22 | c.835C>G | p.P279A | 279 | pathogenic | 2 |
| GRCh37 | 22 | c.842C>T | p.P281L | 281 | pathogenic | 4 |
| GRCh37 | 22 | c.841C>A | p.P281T | 281 | pathogenic | 4 |
| GRCh37 | 22 | c.847C>T | p.R283W | 283 | pathogenic | 4 |
| GRCh37 | 22 | c.848G>A | p.R283Q | 283 | pathogenic | 4 |
| GRCh37 | 22 | c.850C>A | p.R284S | 284 | pathogenic | 4 |
| GRCh37 | 22 | c.850C>T | p.R284C | 284 | pathogenic | 4 |
| GRCh37 | 22 | c.851G>A | p.R284H | 284 | pathogenic | 4 |
| GRCh37 | 22 | c.856G>A | p.G286R | 286 | pathogenic | 4 |
| GRCh37 | 22 | c.862A>G | p.T288A | 288 | pathogenic | 4 |
| GRCh37 | 22 | c.874T>C | p.F292L | 292 | pathogenic | 2 |
| GRCh37 | 22 | c.880C>T | p.R294C | 294 | pathogenic | 4 |
| GRCh37 | 22 | c.930G>T | p.E310D | 310 | tolerated | 1 |
| GRCh37 | 22 | c.967G>T | p.V323F | 323 | pathogenic | 4 |
| GRCh37 | 22 | c.1001G>A | p.G334E | 334 | pathogenic | 2 |
| GRCh37 | 22 | c.1019G>A | p.R340Q | 340 | tolerated | 1 |
| GRCh37 | 22 | c.1027G>A | p.A343T | 343 | tolerated | 0 |
| GRCh37 | 22 | c.1054T>C | p.Y352H | 352 | tolerated | 0 |
| GRCh37 | 22 | c.1058A>C | p.E353A | 353 | tolerated | 0 |
| GRCh37 | 22 | c.1061A>G | p.E354G | 354 | tolerated | 1 |
| GRCh37 | 22 | c.1063C>T | p.R355W | 355 | pathogenic | 3 |
| GRCh37 | 22 | c.1069G>T | p.G357C | 357 | pathogenic | 2 |
| GRCh37 | 22 | c.1074C>G | p.F358L | 358 | tolerated | 0 |
| GRCh37 | 22 | c.1097G>A | p.G366D | 366 | pathogenic | 2 |
| GRCh37 | 22 | c.1102G>C | p.D368H | 368 | pathogenic | 2 |
| GRCh37 | 22 | c.1125G>T | p.K375N | 375 | tolerated | 0 |
| GRCh37 | 22 | c.1142C>T | p.A381V | 381 | tolerated | 0 |
| GRCh37 | 22 | c.1174G>A | p.A392T | 392 | pathogenic | 3 |

| | | | | | | |
|---|---|---|---|---|---|---|
| GRCh37 | 22 | c.1181C>A | p.A394D | 394 | pathogenic | 3 |
| GRCh37 | 22 | c.1183G>T | p.V395F | 395 | pathogenic | 4 |
| GRCh37 | 22 | c.1200G>A | p.M400I | 400 | pathogenic | 3 |
| GRCh37 | 22 | c.1204A>G | p.I402V | 402 | tolerated | 1 |
| GRCh37 | 22 | c.1209C>A | p.F403L | 403 | pathogenic | 4 |
| GRCh37 | 22 | c.1210G>A | p.G404R | 404 | pathogenic | 4 |
| GRCh37 | 22 | c.1211G>A | p.G404E | 404 | pathogenic | 4 |
| GRCh37 | 22 | c.1217C>T | p.T406M | 406 | pathogenic | 4 |
| GRCh37 | 22 | c.1235G>A | p.R412H | 412 | pathogenic | 4 |
| GRCh37 | 22 | c.1234C>T | p.R412C | 412 | pathogenic | 4 |
| GRCh37 | 22 | c.1265C>T | p.S422F | 422 | pathogenic | 4 |
| GRCh37 | 22 | c.1274C>A | p.P425H | 425 | pathogenic | 4 |
| GRCh37 | 22 | c.1292A>T | p.E431V | 431 | pathogenic | 4 |
| GRCh37 | 22 | c.1295A>C | p.D432A | 432 | pathogenic | 4 |
| GRCh37 | 22 | c.1296C>A | p.D432E | 432 | pathogenic | 3 |
| GRCh37 | 22 | c.1300G>T | p.G434W | 434 | pathogenic | 4 |
| GRCh37 | 22 | c.1319G>A | p.R440H | 440 | pathogenic | 2 |
| GRCh37 | 22 | c.1341C>A | p.F447L | 447 | pathogenic | 4 |
| GRCh37 | 22 | c.1342G>A | p.V448M | 448 | pathogenic | 3 |
| GRCh37 | 22 | c.1348G>T | p.G450C | 450 | pathogenic | 4 |
| GRCh37 | 22 | c.1366G>A | p.V456M | 456 | pathogenic | 3 |
| GRCh37 | 22 | c.1375C>T | p.H459Y | 459 | pathogenic | 4 |
| GRCh37 | 22 | c.1388T>G | p.V463G | 463 | pathogenic | 4 |
| GRCh37 | 22 | c.1394C>T | p.A465V | 465 | pathogenic | 4 |
| GRCh37 | 22 | c.1397G>A | p.R466Q | 466 | pathogenic | 4 |
| GRCh37 | 22 | c.1396C>T | p.R466W | 466 | pathogenic | 4 |
| GRCh37 | 22 | c.1403G>A | p.R468H | 468 | pathogenic | 3 |
| GRCh37 | 22 | c.1406G>C | p.W469S | 469 | pathogenic | 4 |
| GRCh37 | 22 | c.1412G>A | p.R471H | 471 | pathogenic | 4 |
| GRCh37 | 22 | c.1420A>G | p.I474V | 474 | pathogenic | 3 |
| GRCh37 | 22 | c.1423A>G | p.T475A | 475 | tolerated | 0 |
| GRCh37 | 22 | c.1430C>T | p.A477V | 477 | pathogenic | 3 |
| GRCh37 | 22 | c.1429G>C | p.A477P | 477 | pathogenic | 4 |
| GRCh37 | 22 | c.1439G>T | p.R480M | 480 | pathogenic | 2 |
| GRCh37 | 22 | c.1448A>T | p.Q483L | 483 | pathogenic | 3 |
| GRCh37 | 22 | c.1462G>A | p.E488K | 488 | tolerated | 1 |
| GRCh37 | 22 | c.1468G>A | p.A490T | 490 | tolerated | 0 |
| GRCh37 | 22 | c.1477C>T | p.P493S | 493 | tolerated | 0 |
| GRCh37 | 22 | c.1489C>T | p.P497S | 497 | tolerated | 0 |
| GRCh37 | 22 | c.1498G>T | p.A500S | 500 | tolerated | 0 |
| GRCh37 | 22 | c.1510G>A | p.A504T | 504 | tolerated | 0 |
| GRCh37 | 22 | c.1514G>A | p.R505Q | 505 | tolerated | 0 |
| GRCh37 | 22 | c.1517C>T | p.P506L | 506 | tolerated | 1 |

| | | | | | | |
|---|---|---|---|---|---|---|
| GRCh37 | 22 | c.1540C>T | p.R514W | 514 | pathogenic | 4 |
| GRCh37 | 22 | c.1553C>T | p.A518V | 518 | pathogenic | 2 |
| GRCh37 | 22 | c.1559C>T | p.P520L | 520 | pathogenic | 2 |
| GRCh37 | 22 | c.1564G>A | p.E522K | 522 | pathogenic | 2 |
| GRCh37 | 22 | c.1570C>A | p.L524I | 524 | pathogenic | 2 |
| GRCh37 | 22 | c.1582C>A | p.L528I | 528 | pathogenic | 2 |
| GRCh37 | 22 | c.1626G>T | p.E542D | 542 | tolerated | 1 |
| GRCh37 | 22 | c.1633C>A | p.L545M | 545 | pathogenic | 2 |
| GRCh37 | 22 | c.1666A>G | p.S556G | 556 | tolerated | 1 |
| GRCh37 | 22 | c.1678T>C | p.C560R | 560 | tolerated | 1 |
| GRCh37 | 22 | c.1682G>A | p.R561H | 561 | pathogenic | 4 |
| GRCh37 | 22 | c.1681C>A | p.R561S | 561 | pathogenic | 4 |
| GRCh37 | 22 | c.1692G>T | p.Q564H | 564 | tolerated | 1 |
| GRCh37 | 22 | c.1699C>A | p.R567S | 567 | tolerated | 1 |
| GRCh37 | 22 | c.1705T>C | p.Y569H | 569 | pathogenic | 3 |
| GRCh37 | 22 | c.1711G>A | p.E571K | 571 | pathogenic | 2 |
| GRCh37 | 22 | c.1714G>T | p.A572S | 572 | pathogenic | 2 |
| GRCh37 | 22 | c.1720G>A | p.V574M | 574 | pathogenic | 3 |
| GRCh37 | 22 | c.1735G>A | p.V579M | 579 | pathogenic | 4 |
| GRCh37 | 22 | c.1757C>T | p.A586V | 586 | pathogenic | 3 |
| GRCh37 | 22 | c.1772T>G | p.L591R | 591 | pathogenic | 4 |
| GRCh37 | 22 | c.1801T>A | p.F601I | 601 | pathogenic | 4 |
| GRCh37 | 22 | c.1803C>G | p.F601L | 601 | pathogenic | 4 |
| GRCh37 | 22 | c.1822T>G | p.F608V | 608 | pathogenic | 4 |
| GRCh37 | 22 | c.1852G>A | p.E618K | 618 | pathogenic | 4 |
| GRCh37 | 22 | c.1859T>G | p.L620R | 620 | pathogenic | 4 |
| GRCh37 | 22 | c.1862C>T | p.S621F | 621 | pathogenic | 4 |
| GRCh37 | 22 | c.1880A>G | p.E627G | 627 | pathogenic | 4 |
| GRCh37 | 22 | c.1889G>A | p.R630Q | 630 | pathogenic | 3 |
| GRCh37 | 22 | c.1901A>C | p.Q634P | 634 | pathogenic | 2 |
| GRCh37 | 22 | c.1904C>T | p.P635L | 635 | pathogenic | 4 |
| GRCh37 | 22 | c.1909C>T | p.P637S | 637 | tolerated | 0 |
| GRCh37 | 22 | c.1912C>T | p.R638C | 638 | pathogenic | 4 |
| GRCh37 | 22 | c.1942G>T | p.G648C | 648 | pathogenic | 4 |
| GRCh37 | 22 | c.1949C>A | p.S650Y | 650 | pathogenic | 4 |
| GRCh37 | 22 | c.1951C>G | p.L651V | 651 | pathogenic | 2 |
| GRCh37 | 22 | c.1979A>G | p.E660G | 660 | pathogenic | 2 |
| GRCh37 | 22 | c.1985C>T | p.A662V | 662 | pathogenic | 2 |
| GRCh37 | 22 | c.1991C>T | p.A664V | 664 | tolerated | 0 |
| GRCh37 | 22 | c.2023G>T | p.G675W | 675 | pathogenic | 4 |
| GRCh37 | 22 | c.2032C>T | p.R678W | 678 | pathogenic | 3 |
| GRCh37 | 22 | c.2048C>T | p.A683V | 683 | pathogenic | 2 |
| GRCh37 | 22 | c.2050A>G | p.I684V | 684 | tolerated | 1 |

| | | | | | | |
|---|---|---|---|---|---|---|
| GRCh37 | 22 | c.2059G>A | p.A687T | 687 | pathogenic | 4 |
| GRCh37 | 22 | c.2062C>A | p.R688S | 688 | pathogenic | 4 |
| GRCh37 | 22 | c.2066C>G | p.S689C | 689 | tolerated | 1 |
| GRCh37 | 22 | c.2077G>C | p.E693Q | 693 | pathogenic | 2 |
| GRCh37 | 22 | c.2081C>T | p.A694V | 694 | pathogenic | 4 |
| GRCh37 | 22 | c.2087T>C | p.F696S | 696 | pathogenic | 4 |
| GRCh37 | 22 | c.2090G>A | p.R697Q | 697 | pathogenic | 4 |
| GRCh37 | 22 | c.2100G>A | p.M700I | 700 | pathogenic | 4 |
| GRCh37 | 22 | c.2104G>A | p.E702K | 702 | pathogenic | 2 |
| GRCh37 | 22 | c.2152C>A | p.Q718K | 718 | pathogenic | 2 |
| GRCh37 | 22 | c.2161G>A | p.E721K | 721 | pathogenic | 2 |
| GRCh37 | 22 | c.2169G>A | p.M723I | 723 | pathogenic | 2 |
| GRCh37 | 22 | c.2170C>A | p.L724M | 724 | pathogenic | 2 |
| GRCh37 | 22 | c.2183A>G | p.Y728C | 728 | pathogenic | 4 |
| GRCh37 | 22 | c.2189G>A | p.G730D | 730 | pathogenic | 3 |
| GRCh37 | 22 | c.2212G>A | p.D738N | 738 | pathogenic | 3 |
| GRCh37 | 22 | c.2259C>A | p.N753K | 753 | pathogenic | 4 |
| GRCh37 | 22 | c.2264G>A | p.R755Q | 755 | pathogenic | 4 |
| GRCh37 | 22 | c.2263C>T | p.R755W | 755 | pathogenic | 4 |
| GRCh37 | 22 | c.2264G>T | p.R755L | 755 | pathogenic | 4 |
| GRCh37 | 22 | c.2273C>T | p.A758V | 758 | pathogenic | 2 |
| GRCh37 | 22 | c.2298G>A | p.M766I | 766 | pathogenic | 2 |
| GRCh37 | 22 | c.2306C>T | p.T769M | 769 | pathogenic | 4 |
| GRCh37 | 22 | c.2316C>A | p.N772K | 772 | pathogenic | 4 |
| GRCh37 | 22 | c.2317G>A | p.V773M | 773 | pathogenic | 3 |
| GRCh37 | 22 | c.2348C>T | p.T783M | 783 | pathogenic | 2 |
| GRCh37 | 22 | c.2359G>C | p.D787H | 787 | pathogenic | 4 |
| GRCh37 | 22 | c.2368C>T | p.R790W | 790 | pathogenic | 4 |
| GRCh37 | 22 | c.2373C>G | p.H791Q | 791 | pathogenic | 3 |
| GRCh37 | 22 | c.2390T>A | p.V797E | 797 | pathogenic | 4 |
| GRCh37 | 22 | c.2419C>T | p.P807S | 807 | pathogenic | 3 |
| GRCh37 | 22 | c.2428C>T | p.R810W | 810 | pathogenic | 4 |
| GRCh37 | 22 | c.2432C>T | p.S811L | 811 | tolerated | 0 |
| GRCh37 | 22 | c.2458A>G | p.I820V | 820 | tolerated | 1 |
| GRCh37 | 22 | c.2471T>A | p.L824Q | 824 | pathogenic | 4 |
| GRCh37 | 22 | c.2500G>T | p.A834S | 834 | pathogenic | 2 |
| GRCh37 | 22 | c.2515G>A | p.D839N | 839 | pathogenic | 3 |
| GRCh37 | 22 | c.2520C>G | p.I840M | 840 | pathogenic | 3 |

**a)**

```
LZTR1          SDSVEYLTLNFGPFETVHRWRRLPPCDEFVGARRSKHTVVAYKDAIYVFGGDNG--KTML    58
5A10           -SPEFMAR------TLQGEWMKVEQKGGQVPAPRSSHGIAVIGDKLYCFGGEDPPYESID    53
                .                 .* ::   .  * * **.* :..  * :* ***::   :::

LZTR1          NDLLRFDVKDCSWCRAFTTGTPPAPRY-HHSAVVYGSSMFVFGGYTGDIYSNSNLKNKND    117
5A10           NDLYVFDFNTHTWSIAPANGDVPKTRVLGTRMVAVGTKLYVFGGRNKQ-------LEFED    106
               ***  **.:  :*. * :.*  *  *      *. *:.::**** . :        : :*

LZTR1          LFEYKFATGQWTEWKIEGRL--PVARSAHGATVYSDKLWIFAGYDGNA------RLNDMW    169
5A10           FYSYDTVKEEWKFLTKLDEKGGPEARTFHSMTSDENHVYVFGGVSKGGLNATPFRFRTIE    166
               ::.*. .. :*.  .  ..   * **: *. *  .::::*.* . ..      *:. :

LZTR1          TIGLQDRELTCWEEVAQSGEIPPSCCNFPVAVCRDKMFVFSGQSGAK-------------    216
5A10           AYNIAEGK---WAQLPDPGEDFEKRGMAGFLVVQGKLWVFYGFATANDPKIPTLYGSQDY    223
               : .: : :   * :: : **    .      . * :.*::** * : *:

LZTR1          ITNNLFQFEFKDKTWTRIPTEHLLRGSPPPPQRRYGHTMVAFDRHLYVFGGAADN-----    271
5A10           ESNRVHCYDPATQKWTEVET-----TGFEKPSRRSCFAHAAVGKYIIIFGGEIERDPEAH    278
                :*.:. ::   :.**.: *       .    *.**  .: .*..::: :***  :.

LZTR1          ----TLPNELHCYDVDFQTWEVVQPSSDSEVGGAEVPERACASEEVPTLTYEERVGFKKS    327
5A10           QGPGTLSREGFALDTETLVWERYEGG----------------------------------    304
                   ** .* .. *.:  .**  : .

LZTR1          RDVFGLDFGTTSAKQPTQPASELPSGRLFHAAAVI----SDAMYIFGGTVDNNIRSGEMY    383
5A10           --------------------PIKPSNRGWVASTTTTINGKKGLLVHGGKLMTNERTDEMY    344
                                   **.* : *::.     ...: :.**.: .* *:.***

LZTR1          RFQFSC  389
5A10           FFAVNS  350
                * ...
```

**b)**

```
LZTR1      FSCYPKCTLHEDYGRLWESRQFCDVEFVLGEKEECVQGHVAIVTARSRWLRRKITQARER 60
4J8Z       MVKVPECRLADELGGLWENSRFTDCCLCVAGQE--FQAHKAILAARSPVFSAMFEHEME- 57
           :   *:* * :: * ***. :* *  : :. :*  .*.* **::***  :   : :  *

LZTR1      LAQKLEQEAAPVPREAPGVAAGGARPPLLHVAIREAEARPFEVLMQFLYTDKIKYPRKGH 120
4J8Z       ------------------------ESKKNRVEINDVEPEVFKEMMCFIYTGKAPNLDK-M 92
                                   .    :* *.:.* . *: :* *:**.*     *

LZTR1      VEDVLLIMDVYKLALSFQLCRLEQLCRQYIEASVDLQNVLVVCESAARLQLSQLKEHCLN 180
4J8Z       ADDLLAAADK------YALERLKVMCEDALCSNLSVENAAEILILADLHSADQLKTQAVD 146
           .:*:*   *       : * **: :*.: : :.:.::*.  :   *   . .*** :.::

LZTR1      FVVKESHFNQVIMMKEFER--LSSPLIVEIVRR--  211
4J8Z       FINY--HASDVLETSGWKSMVVSHPHLVAEAERSL  179
           *:    * .:*:  . ::   :* * :*  ..*
```

**c)**

```
LZTR1      GAGAEFCDITLLLDGHPRPAHKAILAARSSYFEAMFRSFMPEDGQVNISIGEMVPSRQAF 60
4J8Z       WENSRFTDCCLCVAGQEFQAHKAILAARSPVFSAMFEHEMEESKKNRVEINDVEP--EVF 58
              .:.* *  * : *:   ********** *.***.  * *. : .:.*.:: *  :.*

LZTR1      ESMLRYIYYGEVNMPPEDSLYLFAAPYYYGFYNNRLQAYCKQNLEMNVTVQNVLQILEAA 120
4J8Z       KEMMCFIYTGKAPNLDKMADDLLAAADKY--ALERLKVMCEDALCSNLSVENAAEILILA 116
           :.*: :** *:.    : :  *:**   *    :**:. *:: *  *::*:*. :**  *

LZTR1      DKTQALDMKRHCLHIIVHQFTKVSKLPTLRSLSQQLLLDIIDSLASHI     168
4J8Z       DLHSADQLKTQAVDFINYHASDVLETSGWKSMVVS-HPHLVAEAERSL     163
           *  .* ::* :.:.:* :: :.* :    :*:  .   .:: .    :
```

d)

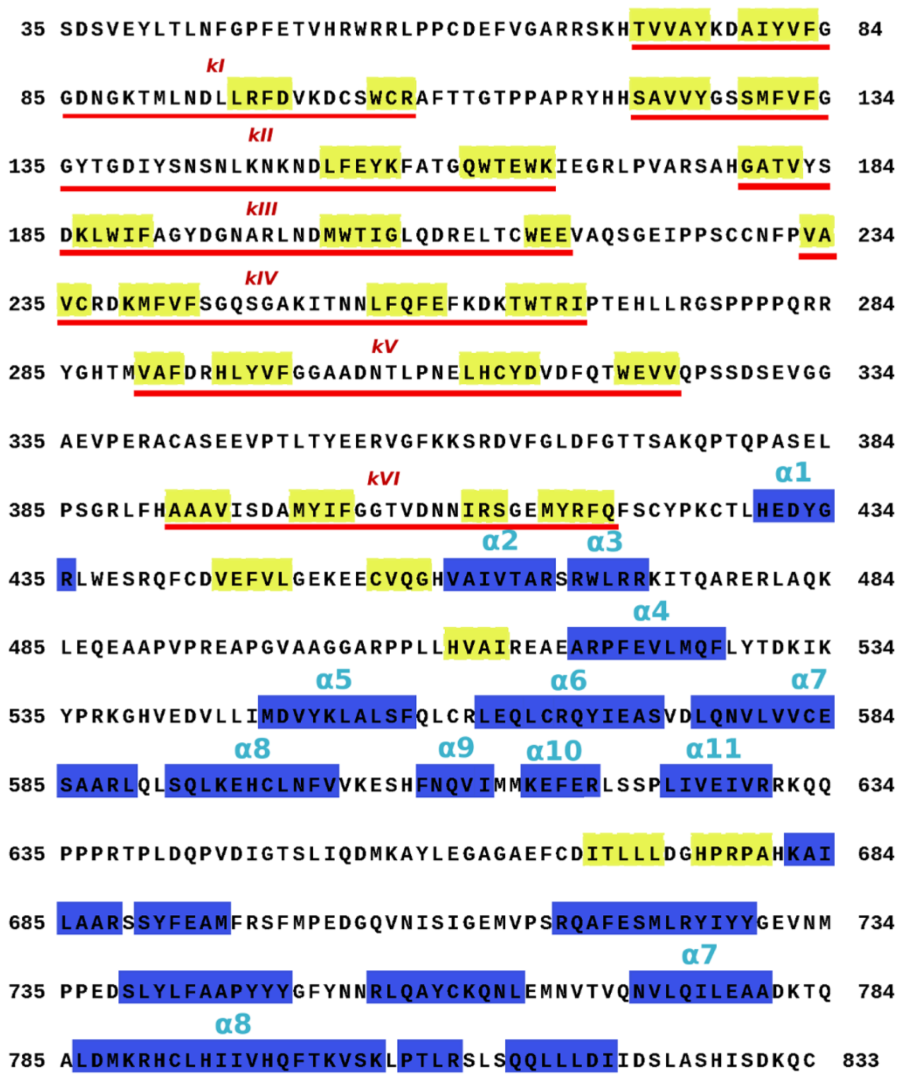


**Figure S1.** Pairwise sequence alignment used for the homology modelling of the human leucine zipper-like trascriptional regulator 1 (LZTR1): **a**) in residual interval 35-423 based on the structure of the Kelch of Thlaspi Arvense Ta-TFP (PDB code 5A10). **b-c**) Pairwise sequence alignments used for the homology modelling of LZTR1 BTB-Back domains in residual interval 429-632 (BTB1) and 651-833 (BTB2), respectively, based on the structure of the human SPOP BTB domain (PDB code 4J8Z). **d**) Full-length LZTR1 sequence along with Secondary Structure prediction and domain composition is shown (α-helices and β-strands are blue and yellow, respectively).

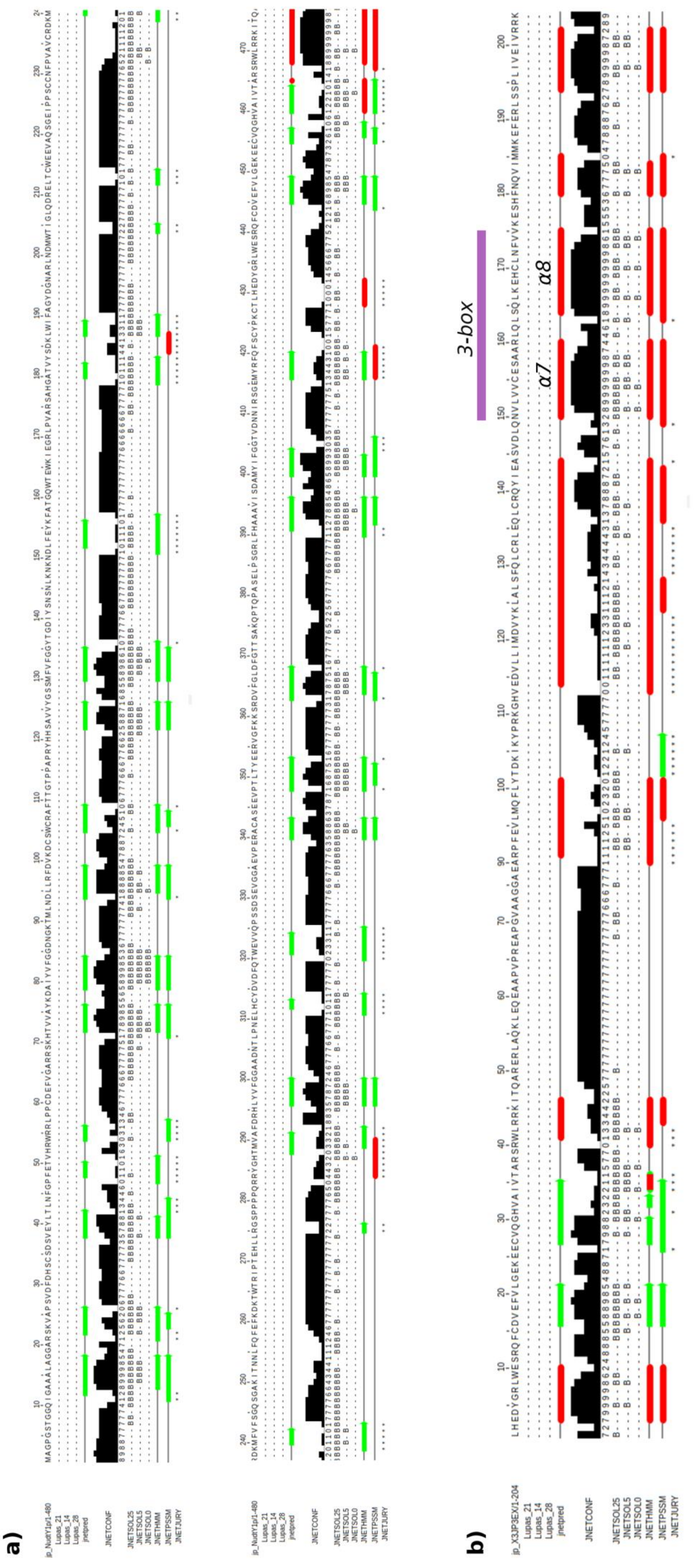
a)
jp_NudtY1p/1-480
Lupas_21
Lupas_14
Lupas_28
jnetpred
JNETCONF
JNETSOL25
JNETSOL5
JNETSOL0
JNETHMM
JNETPSSM
JNETJURY
b)
jp_X3JP3EX/1-204
3-box
α7
α8

**Figure S2.** Jpred Secondary Structure predictions for the human leucine zipper-like trascriptional regulator 1 (LZTR1): **a**) in the residual interval 35-423 and **b**) in the residual interval 429-632 (BTB1). Secondary Structure elements are shown as green arrows (β-strand) and red cylinders (α-helix).

a)

b)

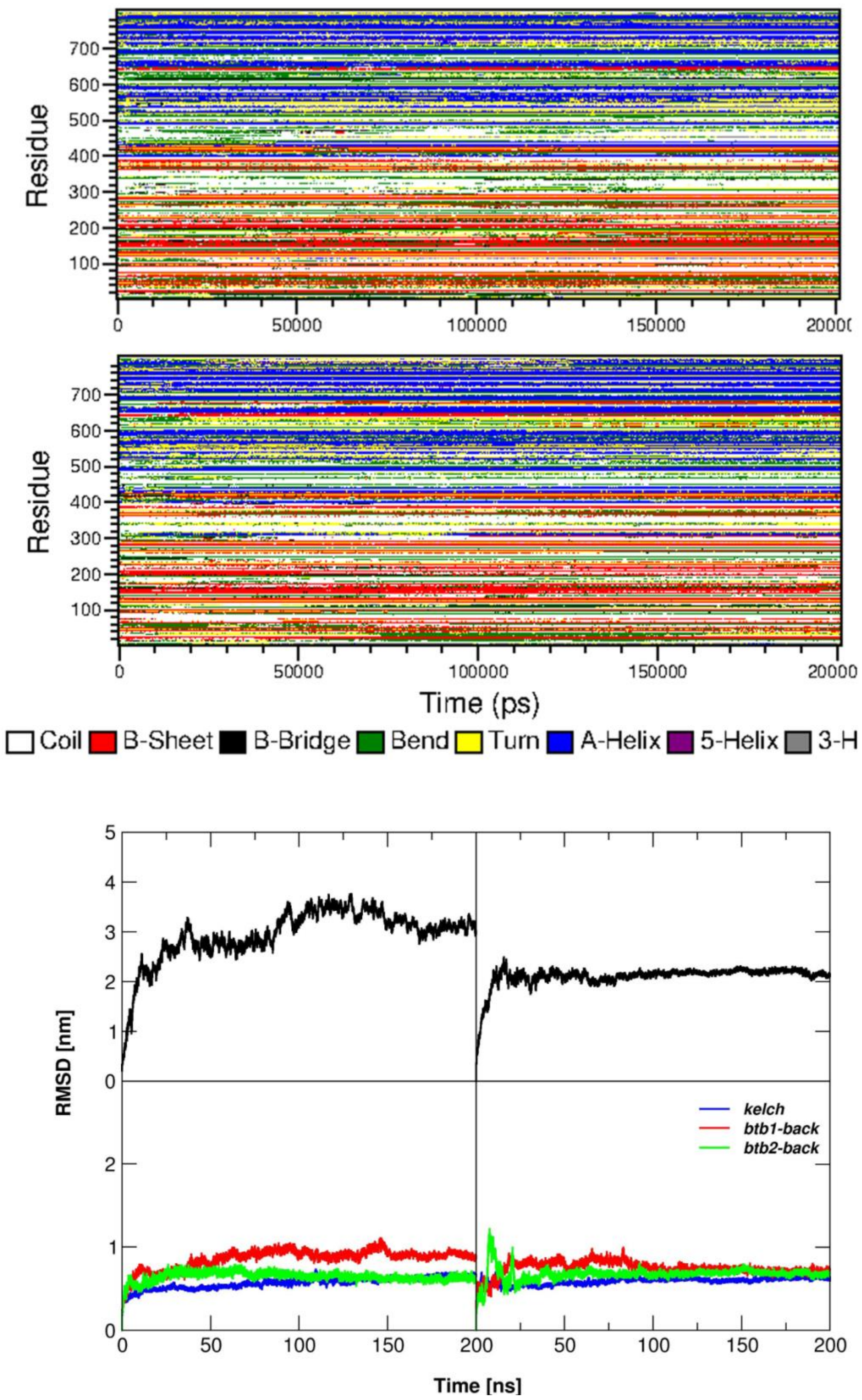


**Figure S3.** LZTR1 structural stability. Secondary structure and Root Mean Square Deviation for the two LZTR1 MD runs. a) Evolution of secondary structure elements and b) full-length RMSD in black lines (top panel) and split per domains (bottom panel). RMSD is calculated on Cα atoms. Note that RMS deviations are given in different scale values.

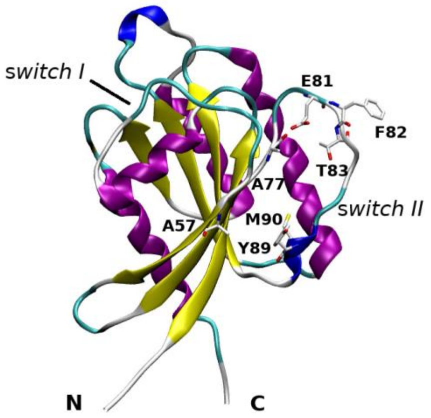


**Figure S4.** 3D structure of the RIT1 protein (PDB access code 4KLZ). Amino acids mutated in NS and cancer are evidenced as white sticks and labeled. Switch I (β2-α1 ) and switch II (β3-α2) responsible for structural transitions between GTP-bound and GDP-bound states are also indicated.

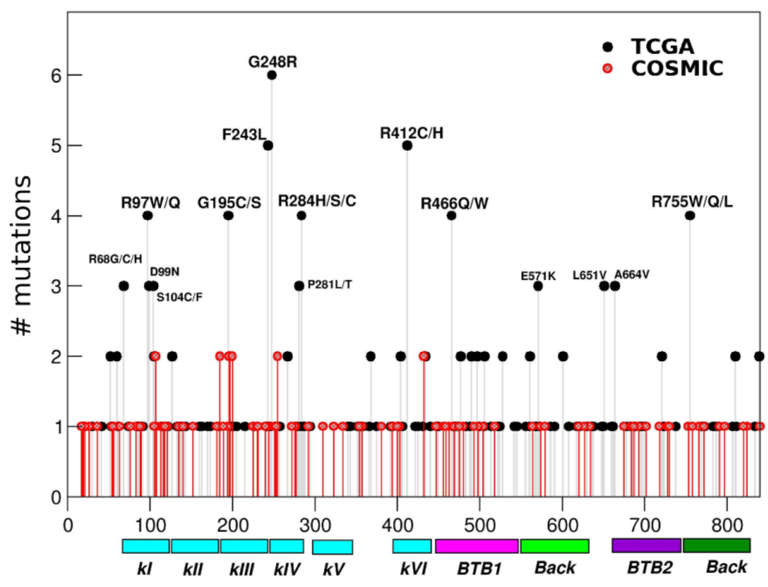


**Figure S5.** Pathogenic mutations found in human LZTR1 gene in Schwannomatosis, Noonan Syndrome and Glioblastoma disorders. x-axis: LZTR1 structural motifs are indicated as colored boxes. Mutations retrieved from TCGA and Cosmic databases are indicated as black and red circles, respectively. For clarity, only for counts ≥ 3 protein variants are reported.

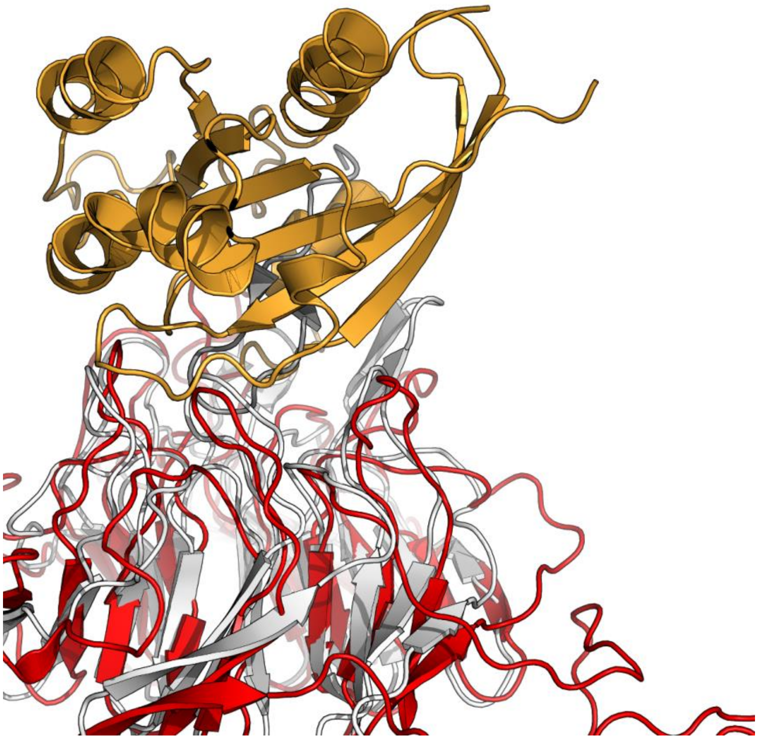

**Figure S6.** Structure superposition of substrate-binding proteins: LZTR1 and RIT1 in red and orange cartoons, respectively. Keap1 and Nrf2 shown in white and grey (pdb 2FLU). Sequence alignment between the two kelch domains is ~ 23%.

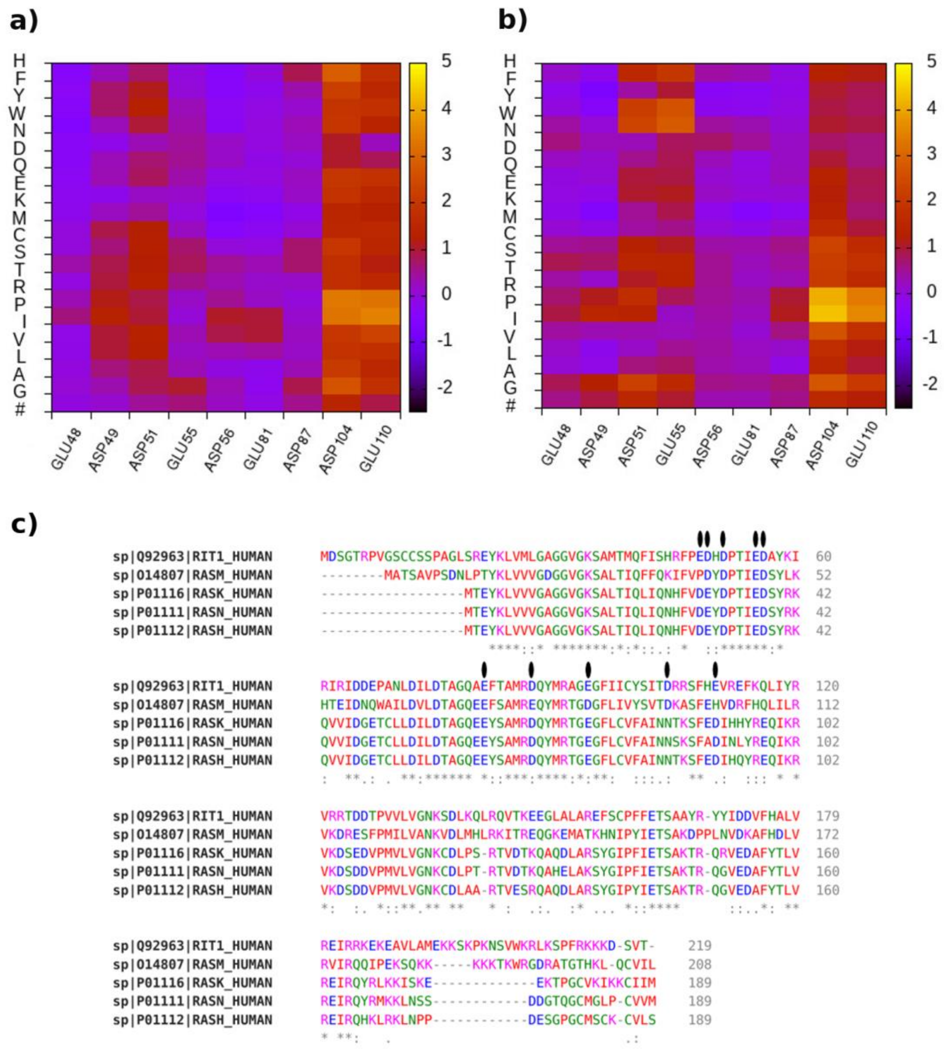


**Figure S7**. **FoldX mutational analysis.** Position scanning is applied to amino acids involved in ionic interactions with LZTR1 as reported in Table 2. Mutagenesis analysis is performed for free RIT1 after equilibration phase (a) and in complex with LZTR1 (b) on the cluster centroid from MD simulations as in Fig 4. C) sequence alignment of human GTPase RAS proteins  is provided. Black ellipses indicate amino acids involved in ionic bonds with LZTR1.

| Donor | Acceptor |
|---|---|
| K70 | D51 |
| R118 | D49 |
| R118 | D49 |
| H120 | D51 |
| Y141 | Q134 |
| Q410 | D56 |
| R412 | D56 |
| Q79 | D304 |
| T83 | D304 |
| A84 | Q305 |
| R86 | Q305 |
| Y89 | D408 |

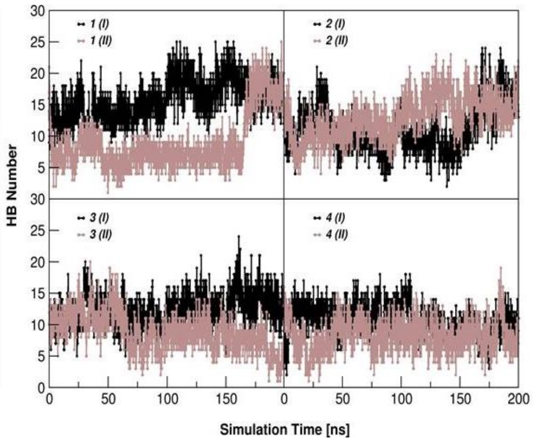


**Figure S8.** Hydrogen bonds made between LZTR1 and RIT1. Hydrogen interactions present in the starting *wt* LZTR1-RIT1 structure after equilibration are listed in the table: red labels are used for amino acids of RIT1 protein. On the right the evolution of hydrogen bonds along MD simulation is plotted for the 2 replicas per complex **1**, **2**, **3** and **4**.

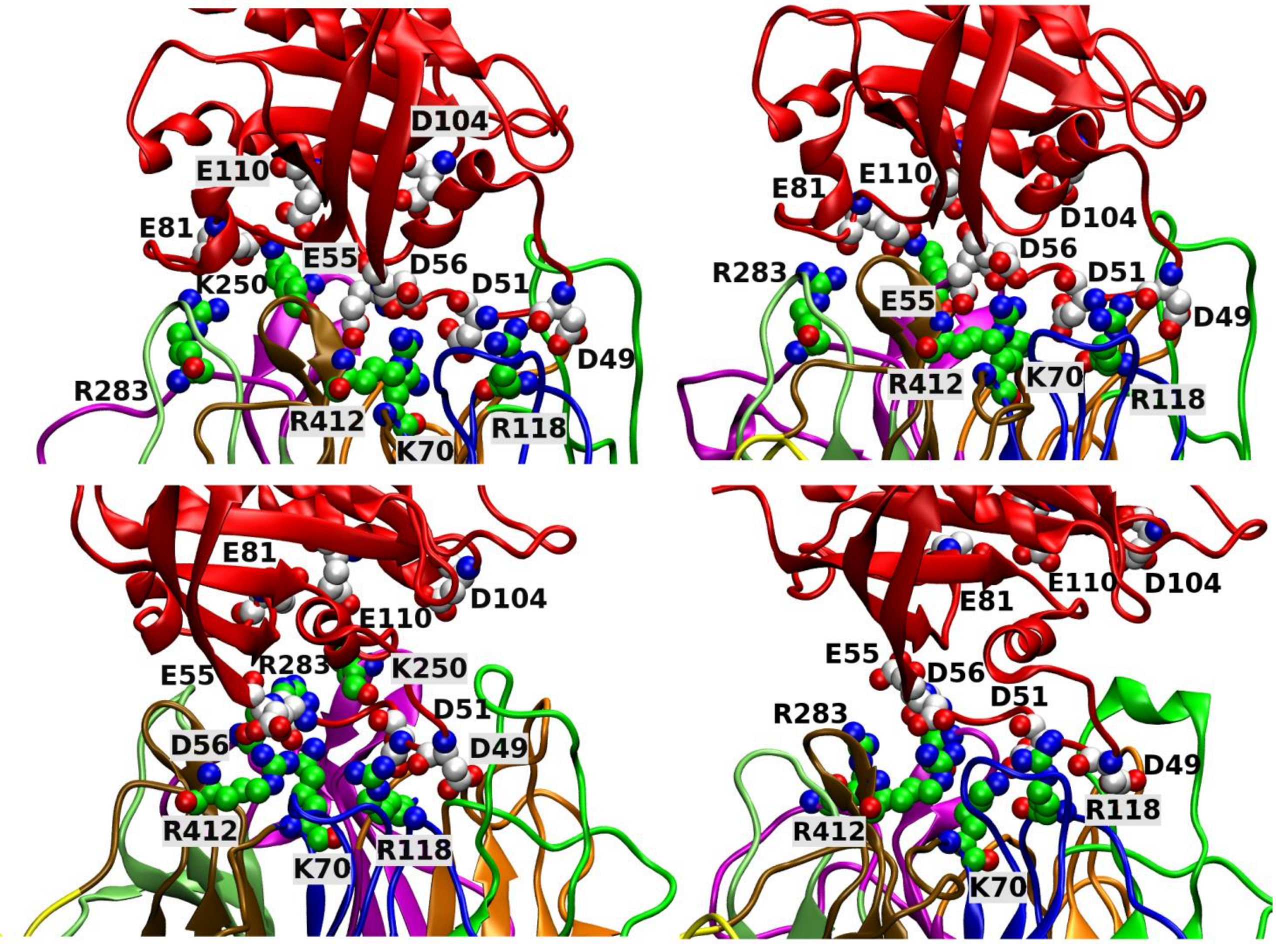

**Figure S9**. **Protein-Protein interaction**. Focus on LZTR1 Kelch domain and RIT1 interacting sites: amino acids from LZTR1 and RIT1 are displayed as green and white CPK, respectively. Four different snapshots taken from cluster analysis carried out on the *wild-type* MD simulations. See table 2 in the main text.

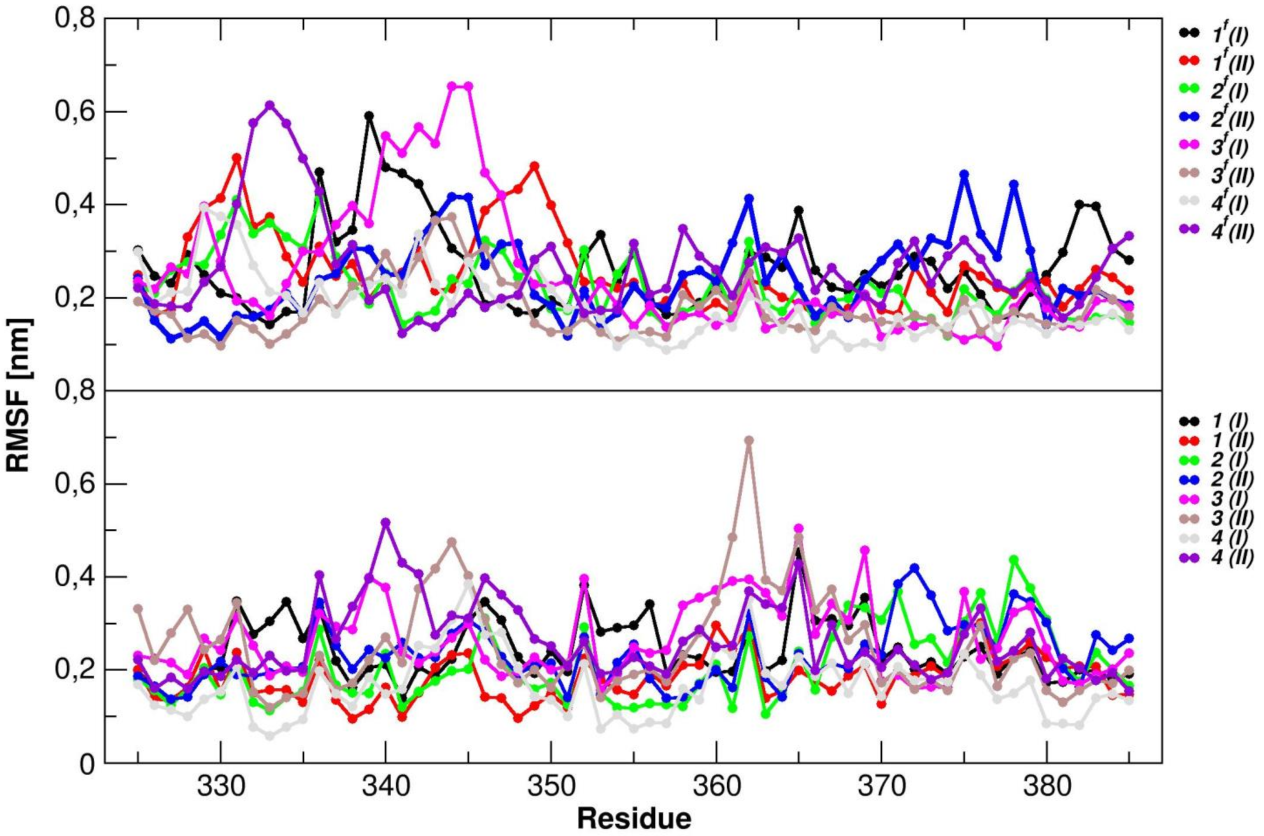


**Figure S10.** Loop RMSF. Average Root Mean Square Fluctuation per 325-385 loop residue. Fluctuations are calculated on Cα atoms along the trajectories (20-200 ns).
